# Structural and functional characterization of MrpR, the master repressor of the *Bacillus subtilis* prophage SPβ

**DOI:** 10.1093/nar/gkad675

**Published:** 2023-08-21

**Authors:** Katharina Kohm, Ekaterina Jalomo-Khayrova, Aileen Krüger, Syamantak Basu, Wieland Steinchen, Gert Bange, Julia Frunzke, Robert Hertel, Fabian M Commichau, Laura Czech

**Affiliations:** FG Synthetic Microbiology, Institute for Biotechnology, BTU Cottbus-Senftenberg, Senftenberg, Germany; FG Molecular Microbiology, Institute for Biology, University of Hohenheim, Stuttgart, Germany; Center for Synthetic Microbiology (SYNMIKRO) and Department of Chemistry, Phillips-University Marburg, Marburg, Germany; Institute of Bio- and Geosciences, iBG-1: Biotechnology, FZ Jülich, Germany; FG Synthetic Microbiology, Institute for Biotechnology, BTU Cottbus-Senftenberg, Senftenberg, Germany; Center for Synthetic Microbiology (SYNMIKRO) and Department of Chemistry, Phillips-University Marburg, Marburg, Germany; Center for Synthetic Microbiology (SYNMIKRO) and Department of Chemistry, Phillips-University Marburg, Marburg, Germany; Max-Planck Institute for Terrestrial Microbiology, Marburg, Germany; Institute of Bio- and Geosciences, iBG-1: Biotechnology, FZ Jülich, Germany; FG Synthetic Microbiology, Institute for Biotechnology, BTU Cottbus-Senftenberg, Senftenberg, Germany; Department of Genomic and Applied Microbiology, Institute of Microbiology and Genetics, Georg-August-University of Göttingen, Göttingen, Germany; FG Synthetic Microbiology, Institute for Biotechnology, BTU Cottbus-Senftenberg, Senftenberg, Germany; FG Molecular Microbiology, Institute for Biology, University of Hohenheim, Stuttgart, Germany; Center for Synthetic Microbiology (SYNMIKRO) and Department of Chemistry, Phillips-University Marburg, Marburg, Germany

## Abstract

Prophages control their lifestyle to either be maintained within the host genome or enter the lytic cycle. *Bacillus subtilis* contains the SPβ prophage whose lysogenic state depends on the MrpR (YopR) protein, a key component of the lysis-lysogeny decision system. Using a historic *B. subtilis* strain harboring the heat-sensitive SPβ c2 mutant, we demonstrate that the lytic cycle of SPβ c2 can be induced by heat due to a single nucleotide exchange in the *mrpR* gene, rendering the encoded MrpR^G136E^ protein temperature-sensitive. Structural characterization revealed that MrpR is a DNA-binding protein resembling the overall fold of tyrosine recombinases. MrpR has lost its recombinase function and the G136E exchange impairs its higher-order structure and DNA binding activity. Genome-wide profiling of MrpR binding revealed its association with the previously identified SPbeta repeated element (SPBRE) in the SPβ genome. MrpR functions as a master repressor of SPβ that binds to this conserved element to maintain lysogeny. The heat-inducible excision of the SPβ c2 mutant remains reliant on the serine recombinase SprA. A suppressor mutant analysis identified a previously unknown component of the lysis-lysogeny management system that is crucial for the induction of the lytic cycle of SPβ.

## Graphical Abstract

**Graphical Abstract:**
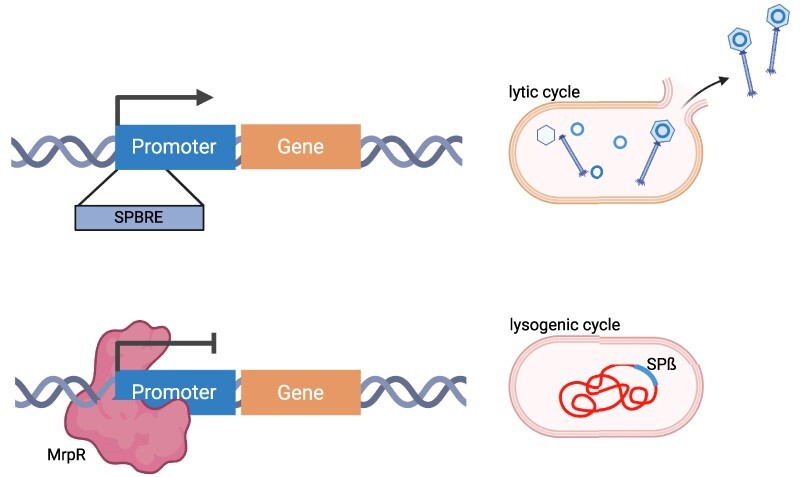

## INTRODUCTION

Phages are bacterial viruses whose replication strictly depends on the host cell. While most host cells are killed after phage infection during the lytic cycle, some phages may enter the lysogenic or temperate life cycle (1,2). In the latter case, the phage genome integrates into the host chromosome and the bacteria carrying the prophage become lysogens. Prophages multiplying together with the host chromosome may enter the lytic cycle through the action of mutagenic agents that elicit a bacterial SOS response via DNA damage (3).

**Figure 1.**
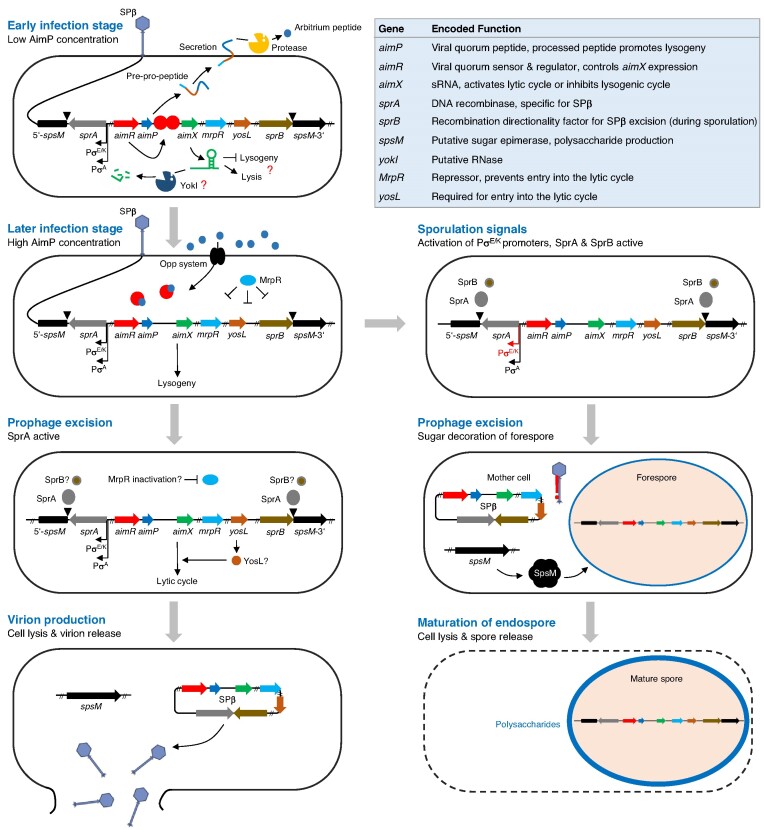
Major steps of the lytic and lysogenic cycles of SPβ. Gene and protein sizes are not drawn to scale. The target of the *aimX* sRNA is unknown and the potential RNase function of YokI remains to be elucidated. The signal that inhibits DNA-binding activity of MrpR and the recombinase catalyzing the excision of the prophage are unknown.

The temperate phage SPβ infects the endospore-forming Gram-positive model bacterium *Bacillus subtilis* and resides as a prophage in the genome of the *B. subtilis* laboratory strain 168 (4). SPβ resembles the *Siphoviridae* morphotype and carries a 130 kb long genome (5). It was discovered about 50 years ago in the *B. subtilis* strain CU1050 which was subjected to chemical mutagenesis and cured of the prophage (3,6,7). Interestingly, cultures of *B. subtilis* that are lysogenic for SPβ release the *S*-linked glycopeptide sublancin, which belongs to the class of antimicrobial natural products named glycocins (8,9). Sublancin inhibits the growth of SPβ-free *B. subtilis* strains by interfering with essential cellular processes such as DNA replication and transcription (8,10). Thus, SPβ is a host-benefitting trait after the transfer to another *B. subtilis* strain lacking the prophage previously (11). Moreover, the presence of SPβ in the genomes of *B. subtilis* strains can be identified based on their antimicrobial properties (8). Like other temperate phages, SPβ may enter either the lytic or the lysogenic cycle. The lytic cycle of SPβ can be induced by treating *B. subtilis* with either *N*-methyl-*N’*-nitro-*N*-nitroso-guanidine or mitomycin C (3). A study focusing on sporulation revealed that the SPβ genome is excised from the mother cell genome during the formation of the spore (Figure 1) (12). The excision process depends on the serine recombinase SprA (SPβ site-specific recombination factor A), whose activity is controlled by the accessory factor SprB (12–14). The excision of the SPβ genome from the host cell genome causes the reconstitution of the *spsM* gene in the mother cell, which is necessary for spore formation but ultimately lyses (Figure 1). The reconstituted *spsM* gene encodes an enzyme involved in sugar decoration of the spore and is thus crucial for spore maturation (12–14). While the *sprA* gene is actively transcribed during vegetative growth, the expression of the *sprB* gene depends on the sporulation-specific sigma factors SigE and SigK (12). Thus, the lack of SprB synthesis during vegetative growth of *B. subtilis* prevents the excision of the SPβ genome. Interestingly, while SPβ resides as a prophage in the spore genome, it is only excised in the mother cell to form the functional *spsM* gene. Moreover, this excision does not result in the formation of infectious phage particles (12).

Recently, it has been demonstrated that the phages of the SPβ group use a small-molecule communication system (termed the ‘arbitrium’ system) to coordinate lysis-lysogeny decisions (Figure 1) (15,16). The core of the arbitrium system consists of the *aimP, aimR* and *aimX* genes encoding the AimP peptide, the transcriptional activator AimR and the non-coding (nc) RNA AimX, respectively (15). In the early stage of infection, the number of phages and the concentration of AimP is low. Apo-AimR binds as a homodimer *via* its helix-turn-helix domain to the operator in the *P_aimX_* promoter and activates transcription of the AimX ncRNA that promotes the lytic cycle (Figure 1) (15,17–20). During the lytic cycle, accumulating AimP is secreted *via* the Sec pathway and processed by extracellular proteases. The resulting hexapeptide is transported into the cell by the Opp oligopeptide transport system (21). The formation of an AimP-hexapeptide/AimR complex inhibits the DNA-binding activity of AimR and promotes the switch to the lysogenic cycle (15). While the molecular details of the AimP-hexapeptide/AimR complex have been elucidated (17–20,22–24), the components that act downstream of the AimX ncRNA remain to be identified (Figure 1). However, the AimP-hexapeptide-dependent control of AimR is crucial to prevent the killing of the entire bacterial population by SPβ, thereby ensuring long-term prophage maintenance.

Two recent studies uncovered several novel players of the SPβ lysis-lysogeny decision system (25,26). For instance, a suppressor analysis with an SPβ *aimR* mutant, which generates more lysogenic cells, revealed that the spontaneous inactivation of the *yopN* gene, encoding a protein of unknown function, partially restores the control of lysis-lysogeny (25). The same studies identified MrpR (formerly YopR) as the master repressor of the lytic cycle of SPβ (25–27). Although it is unclear how AimR, YopN and MrpR interact with each other, it has been proposed that YopN stimulates the repressor activity of MrpR (25). Furthermore, a recent comparative genome analysis identified *mrpR, yopQ, yopP, aimR* and *yokI* as the core genes of SPβ-like phages that are most likely transcriptionally active during the lysogenic cycle (26). The inactivation of the *yokI* gene indeed verified its role in the lysis-lysogeny decision system of SPβ because the *yokI* mutant released about four times more virions into the supernatant as compared to the parental strain (26). It has been hypothesized that YokI could act as an RNase that prevents the accumulation of the AimX ncRNA, thereby hindering its entry into the lytic cycle (Figure 1) (26).

The above-mentioned prophage-free *B. subtilis* strain CU1050 also served as a host to isolate the ‘historic’ SPβ mutant variants c1 and c2 that likely acquired mutations in genes of the lysis-lysogeny decision system (28,29). The c1 mutant had an apparent plaque phenotype and did not lysogenize *B. subtilis*. By contrast, the c2 mutant proved to be temperature-sensitive and entered the lytic cycle when the temperature was shifted to 50°C for a few min. It has been suggested that c1 and c2 are alleles of the same gene encoding a repressor of SPβ like other temperate phages (29). However, the mutations in the SPβ c1 and c2 mutants and their impact on the lysogeny management system have not been identified and studied to date (30). Furthermore, previous *in silico* searches for regulator binding sites identified the SPbeta repeated element (SPBRE) at various intergenic regions within the SPβ genome (31). Yet, the true role of this putative regulatory element and the protein capable of binding to SPBRE has not been discovered.

Here, we demonstrate that the heat-sensitive SPβ c2 phenotype is due to a single nucleotide exchange in the *mrpR* gene, rendering the encoded MrpR^G136E^ repressor temperature-sensitive. Genetic complementation studies revealed that the wild-type MrpR protein is dominant over the MrpR^G136E^ mutant. Thermal shift assays and biochemical analyses uncovered that the G136E exchange renders MrpR less stable and reduces its affinity to bind DNA *in vitro*. Structural and functional characterization of MrpR revealed that the protein is a DNA-binding protein that has an overall fold like tyrosine recombinases but lacks crucial amino acid residues to function as a recombinase enzyme. Genome-wide profiling of MrpR binding using chromatin affinity purification and sequencing (ChAP-Seq) revealed its association with the SPBRE in the genome of the SPβ prophage. Moreover, cell lysis occurred after heat shock in strains lacking either serine recombinase SprA or its accessory factor SprB, while SprB was also dispensable for the release of infectious particles. Finally, a genetic suppressor analysis with a *B. subtilis* strain carrying the SPβ c2 *mrpR* G407A allele identified the YosL protein as a novel player in the lysis-lysogeny management system. Further analysis showed that the presence of *yosL* seems to be crucial for the lytic cycle of SPβ. The current model of the SPβ lysis-lysogeny management system is discussed.

## MATERIALS AND METHODS

### Reagents

Primers used in this study were purchased from Sigma-Aldrich and are listed in Supplementary Table S1. Chromosomal DNA was isolated from *B. subtilis* using the peqGOLD Bacterial DNA Kit (VWR). Plasmid DNA was isolated using the Nucleospin Extract Kit (Macherey-Nagel), Gene Jet Mini Prep Kit (Thermo Scientific), or Monarch^®^ Plasmid Miniprep (NEB). DNA fragments that were generated by the polymerase chain reaction (PCR) were purified using the PCR Purification Kit (Qiagen), Monarch^®^ PCR & DNA Cleanup Kit (NEB), or Gene Jet gel extraction kit (Thermo Scientific). Phusion DNA polymerase, restriction enzymes and T4 DNA ligase were purchased from Thermo Scientific, NEB, or Promega and used according to the manufacturer's instructions (32). Miscellaneous chemicals and media were purchased from Sigma-Aldrich, Carl Roth and Becton-Dickinson. Plasmids were sequenced by the SeqLab Sequence Laboratories (Microsynth).

### Bacterial strains, media and growth conditions

Bacterial strains are listed in Supplementary Table S2. *Escherichia coli* strains were grown in lysogeny broth (LB) at 37°C with constant shaking at 200 rpm or on LB agar plates. Unless otherwise stated, *B. subtilis* was grown in LB or sporulation (SP) medium at 37°C (33). For phage induction, fresh LB was inoculated to an OD_600_ of 0.1, and the culture was incubated for 2.5 h at 37°C. A heat shock was then applied in a water bath for 10 min at 50°C. To allow phage replication, the culture was further incubated for 2 h at 37°C.

### DNA manipulation, transformation, strain construction and phenotypic analysis

The *Escherichia coli* strains XL1-Blue (Stratagene) and DH10B (34) were used for cloning and transformants were selected on LB agar plates supplemented with ampicillin (100 μg/ml) or kanamycin (50 μg/ml) (Sigma-Aldrich or Carl Roth). *B. subtilis* was transformed with plasmid or chromosomal DNA according to the two-step protocol described previously (35). Transformants were selected on LB plates containing chloramphenicol (5 μg ml^−1^), kanamycin (25 μg ml^−1^), or erythromycin (5 μg ml^−1^). The plasmids constructed in this study are listed in Supplementary Table S2. The plasmid pRH005 was constructed for the deletion of the *cat* gene in strain TS01 using the CRISPR-Cas9 system (36). The gRNA was introduced into the plasmid pJOE8999 using the primer pair RH080/RH081 resulting in plasmid pRH004. Next, DNA fragments flanking the *cat* gene were introduced into the plasmid pRH004 using the primer pairs RH082/RH083 and RH084/RH085. The deletion of the *cat* gene in the strain TS01 was performed as described previously (37). To enable the deletion of the genes of interest with the *ermC* deletion cassette (38), we had to remove the *ermD* resistance cassette in the *B. subtilis* strains KK001 and KK137, giving the strains KK002 (SPβ c2) and KK009 (SPβ), respectively. The plasmid pRH29 was generated for this purpose. The gRNA targeting the *ermD* gene was made by hybridizing the primers RH075 and RH076. The dsDNA fragment was ligated to pJOE8999 (37,39) generating pRH001. The *ermD* deletion cassette was made with the primer pairs RH070/RH112 (flank A) and RH113/RH114 (flank B) and introduced into pRH001, resulting in pRH029. To express the *mrpR*^G407A^ (MrpR^G136E^), *mrpR*^A911T^ (MrpR^Y304F^) and *mrpR*^TAT910-912GCG^ (MrpR^Y304A^) mutant alleles from the *amyE* locus in *B. subtilis*, the plasmids pRH166, pRH180 and pRH181, respectively, were constructed. The *mrpR*^G407A^, *mrpR*^A911T^ and *mrpR*^TAT910-912GCG^ alleles were amplified by PCR using the primer pair PP375/PP376, digested with *Eco*RI and *Bam*HI, and ligated to pAC7 that was cut with the same enzymes. SPβ c2 DNA (*mrpR*^G407A^) as well as the plasmids pEJK27 (*mrpR*^A911T^) and the plasmid pEJK29 (*mrpR*^TAT910-912GCG^) served as templates. The isogenic plasmid pRH167 carrying the wild-type *mrpR* allele was constructed previously (26). The plasmid pRH182 carrying the *mrpR*^AAG505-507GCG^ allele (MrpR ^K169A^) was constructed as follows: Two halves of the plasmid pRH167 were amplified with the primer pairs PP396/HLHR4 and PP397/HLHR16 and purified. The fragments were combined and digested with the enzyme *Bsa*I. The remaining plasmid pRH167 was removed by digesting the sample with *Dpn*I. The purified fragments were then ligated and used to transform *E. coli* XL1-Blue. The integrity of the resulting plasmid pRH182 was verified by control digestion and Sanger sequencing. The expression of the *mrpR* alleles from the plasmids pRH166, pRH167, pRH180, pRH181 and pRH182 is driven by the artificial *P_alf4_* promoter and the ribosome-binding site of the *B. subtilis gapA* that were attached by PCR (40). To express the wild-type *mrpR* and the *mrpR*^G407A^ alleles from the *ganA* locus in *B. subtilis*, we constructed the plasmids pRH174 and pKK005, respectively. The *mrpR* alleles were amplified by PCR using the primer pair PP375/PP376 and introduced into the plasmid pGP888 via the enzymes *Eco*RI and *Bam*HI. The artificial *P_alf4_* promoter for constitutive expression was attached by PCR. To study the role of MrpR on the activity of the *P_yosX_* and *P_aimR_* promoters, we constructed the plasmids pRH170 (*P_yosX_ aphA3*), pRH172 (*P_yosX_ cat*), pRH171 (*P_aimR_ aphA3*) and pRH173 (*P_aimR_ cat*). For this purpose, we amplified the intergenic regions upstream of the *yosX* and *aimR* genes with the primer pairs PP388/PP389 and PP390/PP391 and introduced the fragment into the plasmid pAC5 (*cat*) and pAC7 (*aphA3*) using the restriction sites of *Eco*RI and *Bam*HI. Plasmid pRH168 was constructed for the xylose-dependent *yosL* gene from the *ganA* locus. For this purpose, the *yosL* gene was amplified by PCR with the primer pair PP366/PP367, digested with *Eco*RI and *Bam*HI, and ligated to pGP888 that was cut with the same enzymes. The SPβ and SPβ c2 phages were isolated from overnight cultures of the *B. subtilis* strains 168 and CU1147, respectively, by centrifuging 2 ml of the cultures for 1 min at 10 000 × g. Bacterial cells were removed by filtration and the sterile supernatants were used to infect the *B. subtilis* strain TS01, resulting in the lysogens KK001 (SPβ) and KK137 (SPβ c2). The isolation of lysogens from plaques and the sublancin assay to verify the presence of the prophages were described previously (26,39). For the purification of the MrpR, MrpR^G136E^ and YosL proteins, the underlying gene fragments were amplified by PCR using the primer pairs LC195/LC196 for both versions of MrpR and LC192/193 for YosL and introduced into a pET24d (Novagen) derivative modified for modular cloning via *Bsa*I restriction sites. This resulted in the plasmids pEJK11 (MrpR), pEJK13 (MrpR^G136E^) and pEJK9 (YosL) (Supplementary Table S2). All expression constructs encode a C-terminal hexa-histidine (His_6_) tag fused to the target protein. Proteins derived from *B. subtilis* were produced in *E. coli* BL21 (DE3) (NEB). Deletion of *sprA* and *sprB* in the strain KK002 was achieved by transformation using PCR products that were generated with the primer pairs PPKK001/PPKK002 and PPKK003/PPKK004, respectively, and chromosomal DNAs of the strains BKK21660 and BKK19820 as the template. The plasmid pKK012 for the purification of C-terminally Strep-tagged MrpR from *B. subtilis* was constructed as follows. The *mrpR* allele was amplified by PCR using the primer pair PP370/PP374 and chromosomal DNA of *B. subtilis* and used as a template for a second PCR step with the primer pair PP375/PPKK013. The PCR product was digested *Eco*RI/*Bam*HI and ligated to the plasmid pAC7.

### Site-directed mutagenesis

For the overproduction of MrpR variants with amino acid substitutions, we constructed the plasmids pEJK25 (MrpR^K169A^), pEJK27 (MrpR^Y304F^) and pEJK29 (MrpR^Y304A^) using the Q5 Site-Directed Mutagenesis Kit (New England Biolabs; Ipswich, USA) according to the manufacturer's protocol. The primers for mutagenesis were designed with the NEBaseChanger online tool and are listed in Supplementary Table S1.

### Genome sequencing

Genomic DNA was prepared from 500 μl overnight cultures using the peqGold bacterial DNA mini kit following the instructions of the manufacturer, with the modification of physically opening cells with the TissueLyser II (Qiagen). Purified genomic DNA was paired-end sequenced (2 × 150 bp) (GENEWIZ). Sequence libraries were prepared with the NEBNext Ultra II FS DNA Library Prep 136 kit (New England Biolabs GmbH, Frankfurt, Germany) and sequenced with the NovaSeq6000 (Illumina, San Diego, CA, USA). The sequencing reads were mapped onto the genome of the *B. subtilis* strain SP1 and mutations were identified using the applications bowtie2 and breseq (41,42). SP1 is prototrophic for tryptophan and a derivative of the laboratory strain 168 (43). All identified mutations were verified by PCR and Sanger sequencing.

### Electrophoretic mobility shift assays

EMSAs were carried out to analyze the DNA-binding activity of wild-type MrpR and MrpR^G136E^. Regions containing the P*_aimR_*, and *attR* sites were amplified from *B. subtilis* WT168 chromosomal DNA using the primer pairs LC226/LC289*(dye Dyomics 781 at 5′-end), and LC255*(dye Cy3 modification at 5′-end)/LC256, respectively introducing a fluorescent label at one end of the fragment (Supplementary Table S1). In a binding reaction, 1 pmol (50 nM) of the labeled DNA fragment was mixed with the indicated protein concentrations in EMSA buffer containing 20 mM HEPES-Na (pH 7.5), 200 mM NaCl, 20 mM KCl and 50 μg ml^−1^ hering sperm DNA in a final volume of 20 μl. After incubation of the reaction mixture at RT for 15 min, samples were mixed with 10% glycerol and loaded onto a native 6% (w/v) polyacrylamide gel (in 0.5 × TBE containing 45 mM Tris, 45 mM boric acid and 1 mM EDTA). Samples were separated at 100 V for 90 min and subsequently imaged with the 600 and 700 nm channels of an Odyssey FC Imager (LI-COR Biosciences, Lincoln, US).

### *In vitro* recombination assays

The *in vitro* recombination assays were performed as described previously (14) with minor modifications. Fluorescently labeled DNA substrates were amplified from *B. subtilis* wild-type strain 168 chromosomal DNA using the primer pairs LC255*(Cy3 modification at 5′)/LC256 (610 bp) for *attR* site and EJK54/EJK55*(Cy5 modification at 5′) (242 bp) for *attL* site (Supplementary Table S1). In the assayed reaction, 20 nM of the DNA substrates were mixed with 20 μM MrpR or the indicated MrpR variant in a buffer containing 20 mM HEPES-Na (pH 7.5), 200 mM NaCl and 20 mM KCl in a final volume of 10 μl. The mixture was incubated at 37°C for 60 min. The recombination reaction was stopped by the addition of 0.1% (w/v) SDS and by heat treatment at 85°C for 3 min. Samples were loaded onto a native 6% (w/v) polyacrylamide gel (in 0.5 × TBE containing 45 mM Tris, 45 mM boric acid and 1 mM EDTA). Samples were separated at 100 V for 90 min and subsequently imaged with the 600 and 700 nm channels of an Odyssey FC Imager (LI-COR Biosciences, Lincoln, US).

### β-Galactosidase activity assay

Quantitative studies of *lacZ* expression in *B. subtilis* were performed as described previously (35). Cells were grown at 37°C and 220 rpm in 30 ml of LB medium. The medium was supplemented in 300 ml shake flasks and the cells were grown without additional aeration. Cells were harvested before heat shock and at 30, 60, 90, 120 and 150 min after heat shock or incubation at 50°C. Specific β-galactosidase activities were determined with cell extracts obtained by lysozyme treatment. One unit of β-galactosidase is defined as the amount of enzyme which produces 1 nmol of *o*-nitrophenol per min at 28°C. The BioRad dye-binding assay was used to determine the protein concentrations.

### Overexpression and protein purification

For recombinant overexpression of MrpR-His_6_ (pEJK11), the MrpR-His_6_ variants G136E (pEJK13), K169A (pEJK25), Y304F (pEJK27) and Y304A (pEJK29) and YosL-His_6_ (pEJK9), 2 l of LB medium containing kanamycin (50 μg ml^−1^) and 1% (w/v) lactose for autoinduction of the *P_lac_* promoter driving the expression of the T7 polymerase required for recombinant gene expression were incubated in an aerial shaker for 18 h at 30°C. After harvesting, cells were lysed by a microfluidizer (M110-L, Microfluidics). The lysis buffer contained 20 mM HEPES-Na (pH 8.0), 250 mM NaCl, 20 mM KCl and 40 mM imidazole. Cell debris was then removed by high-speed centrifugation for 20 min at 48 000 × g. All proteins were initially purified by nickel ion affinity chromatography and eluted with lysis buffer containing 250 mM imidazole. Further purification using HiTrap Heparin HP columns (Cytiva, Marlborough, USA) was performed according to the manufacturer's protocol. The eluted proteins were concentrated by centrifugation (3 or 10 kDa MWCO) and further polished by size-exclusion chromatography on an S200 XK16 column (for MrpR and variants) or S75 XK16 column (for YosL) (Cytiva, Marlborough, USA) with size-exclusion chromatography (SEC) buffer consisting of 20 mM HEPES-Na (pH 7.5), 200 mM NaCl and 20 mM KCl. Purified proteins were analyzed for the presence of bound nucleotides by agarose gel electrophoresis and analytical size-exclusion chromatography.

### Analytical size-exclusion chromatography

For the analytical SEC, purified MrpR and MrpR variants were diluted in a buffer containing 20 mM HEPES-Na (pH 6.37), 200 mM NaCl and 20 mM KCl to a final concentration of 100 μM. 100 μl were then injected at 4°C onto a pre-equilibrated S200 300/10 GL analytical size-exclusion column (Cytiva, Marlborough, USA) on an ÄKTA system (UNICORN 7.6; Cytiva). For size calibration, a mixture of standard proteins [thyroglobulin (660 kDa), ferritin (474 kDa), aldolase (160 kDa), conalbumin (76 kDa), ovalbumin (43 kDa) and ribonuclease A (13.7 kDa)] was used according to the manufacturer's protocol (Cytiva, Marlborough, USA). Data were plotted using GraphPad Prism (GraphPad Prism Corp. San Diego, USA).

### Thermal shift assays using nanoDSF

To assess the protein folding and thermal stability of MrpR and its variants, thermal shift assays were performed with nanoDSF integrated in the Prometheus NT.48 (Nano-Temper Technologies GmbH, Germany). Protein solutions of 75–100 μM of MrpR WT, MrpR^G136E^, MrpR^K169A^, MrpR^Y304A^ and MrpR^Y304F^ were soaked into a Prometheus NT.48 Series nanoDSF Grade Standard Capillaries and analyzed using PR. ThermControl software. Data were evaluated and plotted using GraphPad Prism (GraphPad Prism Corp., San Diego, USA). The normalized data were fitted to Boltzmann sigmoidal equation. The *T*_m_ values correspond to the inflection point of the sigmoidal curve (44,45).

### Isothermal titration calorimetry

Ligands and proteins (purified wild-type MrpR and MrpR^G136E^ and MrpR^Y304F^ variants) were diluted with a buffer containing 20 mM HEPES-Na (pH 7.5), 200 mM NaCl and 20 mM KCl. The DNA ligand (*aimR* promotor) was obtained by the hybridization of LC292/LC293 primers at 95°C for 2 min. The sample cell was filled with DNA (*aimR* promotor) at a nominal concentration of 20 μM. The MrpR proteins were placed in the syringe and their concentrations were predetermined by absorbance at 280 nm to saturate the DNA sample during the titrations. All the measurements were performed at 25°C with the instrument MicroCal PEAQ-ITC (©Malvern Panalytical) with a method consisting of 13 injections (first 0.4 μl, and the rest 3 μl each) and 150 s of spacing. The raw data were processed with the MicroCal PEAQ-ITC Analysis Software (Malvern Panalytical) using the ‘one set of sites’ models.

### Crystallization and structure determination

Crystallization was performed by the sitting drop vapor diffusion method at 20°C in 250 ml drops consisting of equal parts of protein and precipitation solutions. Protein solutions of 480 μM MrpR-His_6_ were used for crystallization. A total of 384 different conditions were included in the screen (JCSG Core Suite I-IV, Qiagen) and crystals were obtained in 0.2 M disodium tartrate supplemented with 20% (w/v) PEG3350. Prior to data collection, crystals were flash-frozen in liquid nitrogen using a cryo-solution that consisted of mother liquor supplemented with 20% (v/v) glycerol. Data were collected under cryogenic conditions at the European Synchrotron Radiation Facility (Grenoble, France) (46). MxCube3 was used for data collection (https://github.com/mxcube). Data were processed with XDS (version 31 January 2020) and scaled with XSCALE (47). The structure was determined by molecular replacement with PHASER (48), manually built in COOT (Coot Version 0.9.4.1) (49) and refined with PHENIX (50) (Phenix Version 1.17.1–3660 and 1.19). The search model for the MrpR structure was the N-terminal domain (residues 1–105) of the Alphafold2 model (51). Figures were prepared with Pymol (www.pymol.org). Crystallization data collection and refinement statistics are given in Table 2. Structure coordinates and structure factors of MrpR have been deposited under the PDB-ID: 8A0A (https://doi.org/10.2210/pdb8A0A/pdb).

### Hydrogen/deuterium exchange mass spectrometry

Preparation of samples for HDX-MS was aided by a two-arm robotic autosampler (LEAP Technologies), as described previously with minor modifications (52). MrpR protein variants and DNA (where indicated) were employed at concentrations of 25 μM each. Hydrogen/deuterium exchange (HDX) was initiated by 10-fold dilution of MrpR, MrpR^G136E^ or MrpR^Y304F^ in buffer (20 mM HEPES-Na pH 7.5, 20 mM KCl, 200 mM NaCl) prepared in D_2_O. After incubation at 25°C for 10, 30, 100, 1000 or 10000 s, the HDX was stopped by mixing the reaction with an equal volume of quench buffer (400 mM KH_2_PO_4_/H_3_PO_4_, 2 M guanidine–HCl; pH 2.2) temperated at 1°C, and 100 μl of the resulting mixture was injected (loop volume 50 μl) into an ACQUITY UPLC M-Class System with HDX Technology (53). Non-deuterated samples were generated similarly by 10-fold dilution in buffer prepared with H_2_O. The injected HDX samples were washed out of the injection loop with H_2_O + 0.1% (v/v) formic acid at 100 μl min^−1^ flow rate and guided over a column (2 mm x 2 cm) filled with immobilized protease, facilitating proteolytic digestion at 12°C. The resulting peptides were collected on a trap column (2 mm × 2 cm) that was filled with POROS 20 R2 material (Thermo Scientific) kept at 0.5°C. After three min of digestion and trapping, the trap column was placed in line with an ACQUITY UPLC BEH C18 1.7 μm 1.0 × 100 mm column (Waters) and peptides eluted through a gradient of H_2_O + 0.1% (v/v) formic acid (eluent A) and acetonitrile + 0.1% (v/v) formic acid (eluent B) at 60 μl min^−1^ flow rate as follows: 0–7 min/95–65% A, 7–8 min/65–15% A, 8–10 min/15% A. Eluting peptides were guided to a Synapt G2-Si mass spectrometer (Waters) and ionized by electrospray ionization (250°C capillary temperature, 3.0 kV spray voltage). Mass spectra were acquired from 50 to 2000 *m/z* in enhanced high-definition MS (HDMS^E^) (54,55) or high-definition MS (HDMS) mode for non-deuterated and deuterated samples, respectively. Continuous lock mass correction was implemented with [Glu1]-Fibrinopeptide B standard (Waters). During separation of the peptides on the ACQUITY UPLC BEH C18 column, the pepsin column was washed three times by injecting 80 μl of 0.5 M guanidine hydrochloride in 4% (v/v) acetonitrile. Blank runs (injection of H_2_O instead of protein) were performed between each sample. The experiment was conducted twice in triplicates (individual HDX reactions), whereby either immobilized porcine pepsin or a mixture of immobilized protease type XVIII from *Rhizopus* sp. and protease type XIII from *Aspergillus saitoi* were employed for proteolytic digestion. Peptides were identified and evaluated for their deuterium incorporation with the software ProteinLynx Global SERVER 3.0.1 (PLGS) and DynamX 3.0 (both Waters) as described (52). Peptides were identified with PLGS from the non-deuterated samples acquired with HDMS^E^ employing low energy, elevated energy and intensity thresholds of 300, 100 and 1000 counts, respectively, and matched using a database containing the amino acid sequence of MrpR, porcine pepsin and their reversed sequences with search parameters as follows: Peptide tolerance = automatic; fragment tolerance = automatic; min fragment ion matches per peptide = 1; min fragment ion matches per protein = 7; min peptide matches per protein = 3; maximum hits to return = 20; maximum protein mass = 250 000; primary digest reagent = non-specific; missed cleavages = 0; false discovery rate = 100. For quantification of deuterium incorporation with DynamX, the data obtained from pepsin or fungal protease digestion were combined, and peptides had to fulfil the following criteria: Identification in at least 2 of 3 non-deuterated samples for either protease digestion protocol; the minimum intensity of 10 000 counts; maximum length of 30 amino acids; minimum number of products of two; maximum mass error of 25 ppm; retention time tolerance of 0.5 min. All spectra were manually inspected and omitted, if necessary, e.g. in case of low signal-to-noise ratio or the presence of overlapping peptides disallowing the correct assignment of the isotopic clusters. Residue-specific deuterium uptake from peptides identified in the HDX-MS experiments was calculated with the software DynamX 3.0 (Waters). In the case that any residue is covered by a single peptide, the residue-specific deuterium uptake is equal to that of the whole peptide. In the case of overlapping peptides for any given residue, the residue-specific deuterium uptake is determined by the shortest peptide covering that residue. Where multiple peptides are of the shortest length, the peptide with the residue closest to the peptide C-terminus is utilized. Raw data of deuterium uptake by the identified peptides and residue-specific HDX are provided in Supplemental Dataset.

### Preparation of ChAP-seq samples

The samples for ChAP-Seq analyses were prepared as described previously with the following modifications (56). 12 ml LB overnight precultures of KK152 were used to inoculate 1 l LB main cultures to an OD_600_ of 0.1 and the cells were harvested after 2.5 h of cultivation (OD_600_ of approximately 1.0) at 37°C. After washing the cells with LB, the cells were resuspended in 10 ml LB with 1% (v/v) formaldehyde. Phenylmethylsulfonyl fluoride (PMSF) at a final concentration of 0.5 mM served as a protease inhibitor. Cell disruption was achieved by three passages through a French Press cell at 16.000 PSI. The chromosomal DNA of the lysates was sheared by sonication (2 × 20 s). Cell debris was first removed by centrifugation at 5000 x g for 20 min and then ultra-centrifuged for 1 h at 40 000 rpm (rotor Ti60) both steps at 4°C. The MrpR-Strep protein was isolated using the Streptactin-Strep-tag affinity purification procedure according to the manufacturer's protocol (IBA, Göttingen, Germany). Briefly, the supernatant was applied to an equilibrated 1 ml Strep-Tactin^®^-Sepharose^®^ column. It was subsequently washed with 5 x with 2.5 of the column volume using buffer W (100 mM Tris–HCl, 1 mM EDTA, 150 mM NaCl) and the proteins were eluted with 4 × 0.5 of the column volume with buffer W containing 2.5 mM d-desthiobiotin. 100 μl samples were taken for analysis by SDS page.

### ChAP-seq—sequencing and peak analysis

Isolated DNA fragments were used for library preparation. Indexing was performed using the TruSeq DNA PCR-free sample preparation kit (Illumina, Chesterford, UK) according to the manufacturer's instructions, without the DNA size selection steps. Libraries were consequently quantified using KAPA library quant kit (Peqlab, Bonn, Germany), normalized for pooling and sequenced using the MiSeq device (Illumina) (paired-end sequencing, read length: 2 × 150 bases). Data analysis and base calling were performed with the Illumina instrument software. The resulting fastq output files were further processed and modified according to Keppel *et al.* (57). In a first step, sequencing data was collapsed for each sample to remove PCR amplification artifacts. Collapsed fastq files were mapped to accession NC_000964.3 as *Bacillus subtilis* subsp*. subtilis* strain 168 reference genome with respective genetic modifications. This was done using Bowtie2 with the following parameters: –ignore-quals –local –very-sensitive-local –rfg 9,5 –rdg 9,5 –score-min L,40,1.2 -k 8 –no-unal –no-mixed –threads 8 -I 40 -X 800 (41,58). The genomic coverage was convoluted with second-order Gaussian kernel and the kernel was truncated at four sigmas and expanded to the expected peak width. The expected peak width was predicted in the following steps: (i) Detection of all peaks higher than three mean coverage. (ii) Detection of points at which coverage dropped below half of the maximal peak height, with the distance between those set as peak width. (iii) The estimated peak width was fixed equal to the median peak width. Convolution profiles were scanned for identification of the regions where the first derivative changes from positive to negative. Each of these regions was determined as a potential peak with an assigned convolution score (convolution with second-order Gaussian kernel centered at the peak position). Filtered peaks were normalized for inter-sample comparisons and the sum of coverages of all detected peaks was negated from the total genomic coverage. This difference was used as normalization coefficient and was divided by peak intensities.

### Western blot analysis

Heterologously produced and purified MrpR-His_6_ and YosL-His_6_ proteins were used to generate rabbit polyclonal antibodies (Dr. Benli, Göttingen). For Western blot analyses, the cells were grown as described for the β-galactosidase activity assay. Proteins were separated by 15% SDS–PAA gels. After electrophoresis, the proteins were transferred to a polyvinylidene difluoride membrane (PVDF, BioRad) by electroblotting. MrpR and YosL were detected with polyclonal antibodies (33). Antibodies were visualized by using anti-rabbit immunoglobulin G-alkaline phosphatase secondary antibodies (Promega) and the CDP-star detection system (Roche Diagnostics) as described previously (33).

## RESULTS

### Verification of the SPβ c2 phenotype and generation of lysogens in a prophage-free strain

**Figure 2.**
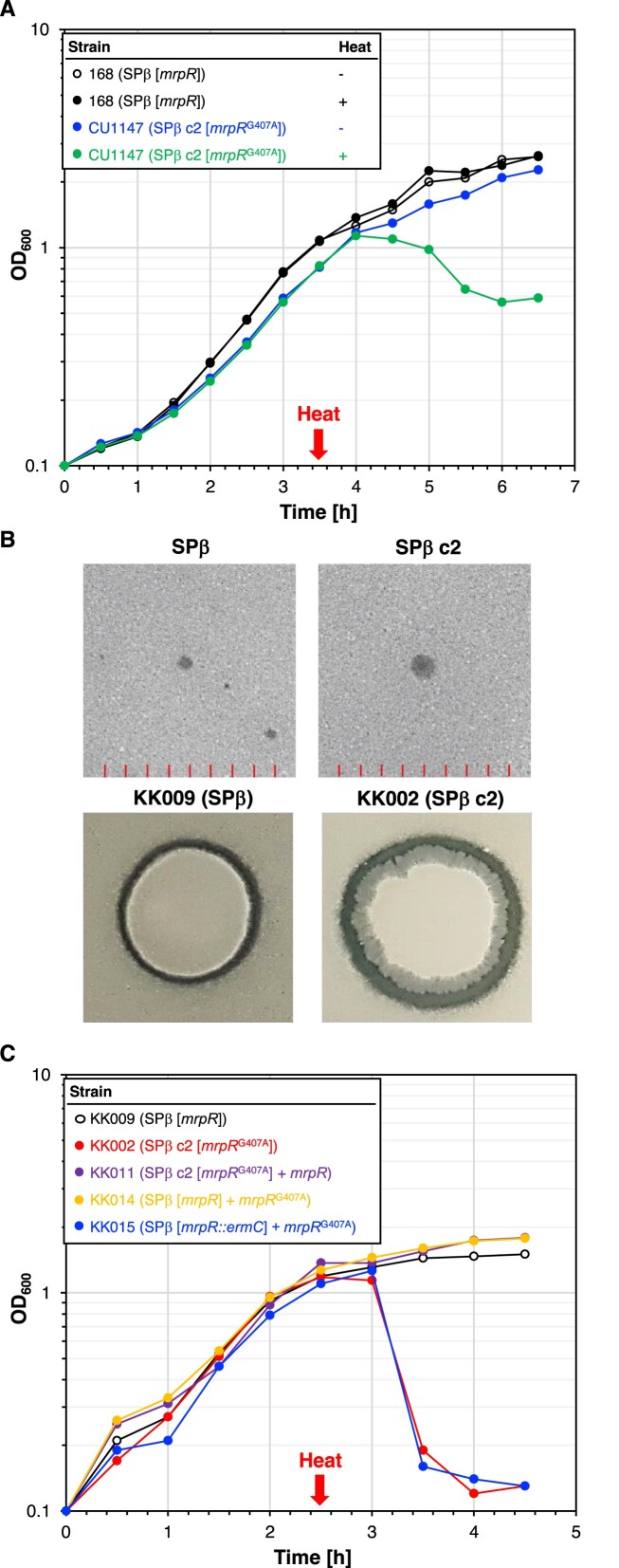
The temperature-sensitive SPβ c2 prophage produces sublancin and senses heat shocks due to a mutation in the *mrpR* gene. (**A**) Heat shock experiment to assess the temperature sensitivity of the SPβ c2 prophage in the historical *B. subtilis* strain CU1147. Cultures of the strains 168 (SPβ [*mrpR*], control) and CU1147 (SPβ c2 [*mrpR*^G407A^]) were grown for 2.5 h at 30°C, heat shocked at 50°C for 10 min and further cultivated at 30°C. The gene marked with square brackets is present in the phage genome. The optical density at a wavelength of 600 nm (OD_600_) was monitored over time. Further induction experiments revealed 37°C as the growing temperature and 10 min heat shock as the best to induce SPβ c2. Single points from one representative of three replicates are shown. (**B**) Plaque assay (upper panel) with SPβ and SPβ c2 lysates and sublancin assay with the strains KK009 and KK002 carrying SPβ and SPβ c2 prophages, respectively (see Materials and Methods). **C**. Heat shock experiments show that the *mrpR*^G407A^ allele causes the temperature-sensitive phenotype of the SPβ c2 prophage. The indicated strains were heat shocked as described in the Materials and Methods section. The gene marked with square brackets is present in the phage genome. The OD_600_ was monitored over time. The strains KK011, KK014 and KK015 express the *mrpR/mrpR*^G407A^ alleles from the *amyE* locus.

To verify the heat-sensitive phenotype of the historical *B. subtilis* strain CU1147 carrying the SPβ c2 prophage, we performed a heat shock experiment (28,29). For this purpose, cultures containing exponentially growing cells of CU1147 were shifted from 37°C to 50°C for 5 min as described previously (29). As shown in Figure 2A, only the cells of CU1147 started to lyse 1 h after heat treatment, confirming the presence of the SPβ c2 in the genome of CU1147 and the heat-sensitive phenotype of the prophage. To explore the underlying genotype of the SPβ c2 phage mutant and to prevent potential crosstalk between SPβ c2 and other prophages and prophage-like elements, we choose the *B. subtilis* strain TS01 as the new host. The TS01 strain is a descendant of 168, lacking the prophages SPβ and PBSX, the prophage-like elements prophage 1, prophage 3 and *skin*, as well as the *pks* polyketide synthesis operon (37,59,60). New lysogens carrying SPβ and SPβ c2 designated as KK009 and KK002, respectively, were isolated from plaques of TS01-infected cells (Figure 2B, upper panel). Further experiments revealed that a cultivation temperature of 37°C and a 10 min-long heat shock are best suited to induce the lytic cycle of SPβ c2 [29]. The sublancin assay indicated the presence of the prophages and the subsequent heat shock experiment confirmed that the heat-induced cell lysis was caused by SPβ c2 (Figure 2B, lower panel; Figure 2C).

### The MrpR^G136E^ variant confers heat induction of the SPβ c2 prophage

Previously, it has been suggested that the c2 allele encodes a repressor of the lytic cycle of SPβ (29). However, the mutation causing the c2 phenotype remained unknown. To identify the mutation(s) conferring heat inducibility of SPβ c2, we re-sequenced the SPβ c2 genome. Sequence variations were identified through a direct alignment with the SPβ genome of the *B. subtilis* laboratory strain SP1 (43). We identified four sequence variations in the *yorN*-*yorM* intergenic region and in the *yoqA, mrpR* and *yokI* genes. The mutation in the *yorN*-*yorM* intergenic region could affect the transcription of the *yorN* gene of unknown function. The remaining three genes contain nonsynonymous mutations (Table 1). While YoqA shares no similarity to any known protein domains, YokI and MrpR share sequence similarities with ribonucleases and DNA-breaking-rejoining proteins, respectively. We speculated that the base exchange G407A in the coding region of *mrpR* is responsible for the heat sensitivity of SPβ c2. To test this idea, we fused the constitutively active synthetic *P_alf4_* promoter (40) and the ribosome binding site of the *B. subtilis gapA* gene to the *mrpR* wild-type and the *mrpR*^G407A^ alleles and integrated the constructs into the *amyE* locus of the strains KK002 (SPβ c2 [*mrpR*^G407A^]) and KK009 (SPβ [*mrpR*]), respectively. The resulting strains were designated as KK011 (SPβ c2 [*mrpR*^G407A^] *P_alf4_-mrpR*) and KK014 (SPβ [*mrpR*] *P_alf4_-mrpR*^G407A^). Next, we constructed the strain KK015 (SPβ [*mrpR::ermC*] *P_alf4_-mrpR*^G407A^) lacking the native SPβ *mrpR* allele. The following heat shock experiment confirmed that the heat-induced cell lysis of *B. subtilis* was due to the mutation in *mrpR* because the strains carrying only the *mrpR*^G407A^ allele (KK002 and KK015) lysed after the heat shock (Figure 2C). Furthermore, no cell lysis was observed with the strains KK011 (SPβ c2 [*mrpR*^G407A^] *P_alf4_-mrpR*) and KK014 (SPβ [*mrpR*] *P_alf4_-mrpR*^G407A^) that carried both *mrpR* alleles (Figure 2C). Thus, the wild-type MrpR protein is dominant over the MrpR^G136E^ variant. To conclude, the G407A mutation in *mrpR* causes the heat sensitivity of MrpR^G136E^.

**Table 1.** Mutations identified in the SPβ c2 phage

Region	Encoded function	Type of mutation	Coordinates^a^
*yokI*	Putative ribonuclease	G145A (G49S)	2.277.281
*mrpR*	Repressor	G407A (G136E)	2.204.767
*yoqA*	Unknown	T68C (L23P)	2.201.412
*yorN-yorM*	Unknown	ΔAG, 25–24 bp upstream of *yorN*	2.174.763–2.174.762

^a^Coordinates refer to the position in the genome sequence CP058242 of the *B. subtilis* SP1 strain (43).

### Characterization of MrpR and MrpR^G136E^

**Figure 3.**
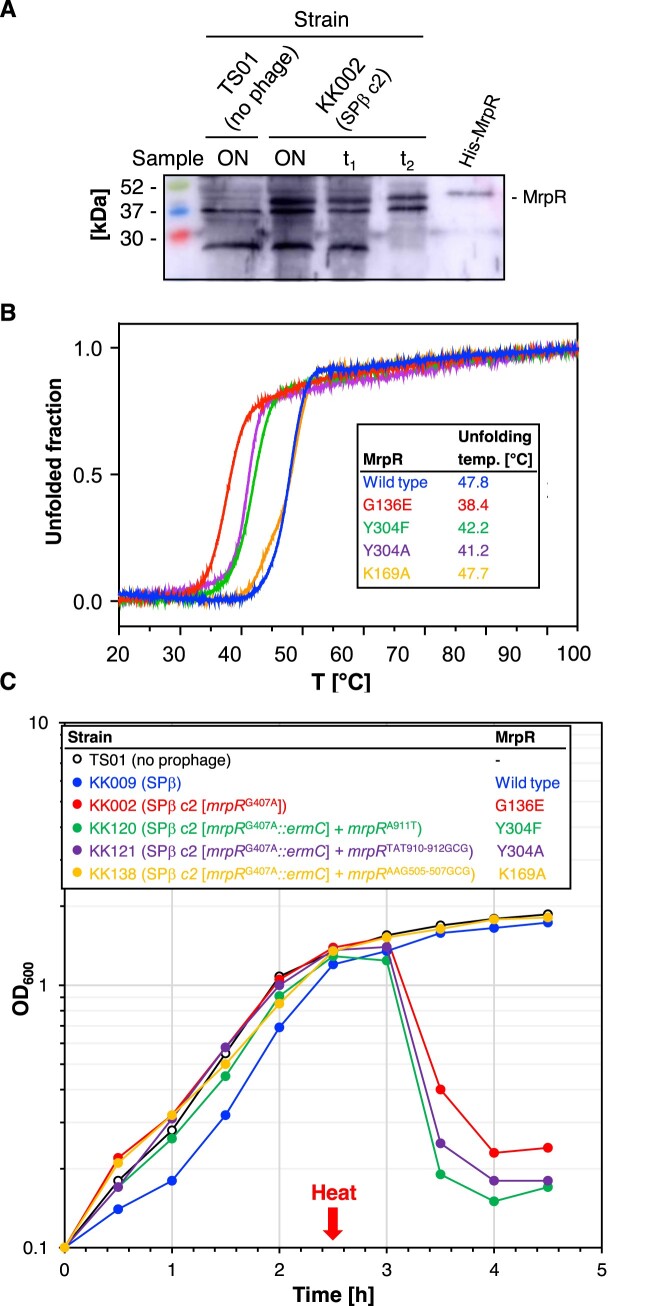
Cellular amounts of MrpR^G136E^ and characterization of MrpR variants. (**A**) Cellular amounts of MrpR^G136E^ were determined by Western blotting. Crude extracts of the indicated strains were isolated from overnight (ON) or exponentially growing LB cultures at timepoints *t*_1_ and *t*_2_, immediately and 30 min after the heat shock (10 min incubation at 50°C), respectively. Purified MrpR-His_6_ (100 ng) served as the control. After electrophoresis in a 12.5% SDS-PAGE and transfer onto a PVDF membrane, MrpR was detected by rabbit polyclonal antibodies raised against MrpR-His_6_. 20 mg of crude extracts were applied. (**B**) Analysis of purified MrpR wild-type (blue), MrpR^G136E^ (red), MrpR^Y304F^ (green), MrpR^Y304A^ (purple) and MrpR^K169A^ (orange) stability by nanoDSF. Thermal unfolding curves of MrpR and MrpR variants were assessed in SEC buffer. Changes in the F350/F330 fluorescence ratio are shown and *T*_m_ values are indicated in the table. (**C**) Heat shock experiment to assess the temperature sensitivities of the MrpR wild-type (blue), MrpR^G136E^ (red), MrpR^Y304F^ (green), MrpR^Y304A^ (purple) and MrpR^K169A^ (orange). The indicated strains were heat shocked as described in the Materials and Methods section. The OD_600_ was monitored over time. Single points from one representative of three replicates are shown.

To assess whether the heat shock affects the cellular concentration of the MrpR^G136E^ variant, we performed a Western blot experiment with crude extracts from the strains TS01 (no phage) and KK002 (SPβ c2) that were grown overnight at 37°C. We also analyzed samples from a culture of the strain KK002 that was heat shocked. As expected, the strain TS01 did not synthesize MrpR (Figure 3A). By contrast, the strain KK002 produced a protein corresponding to the molecular weight of MrpR. Moreover, the bacteria produced comparable amounts of the MrpR^G136E^ variant before and after the heat shock. Therefore, the G136E replacement in MrpR does not affect its abundance *in vivo* but potentially its folding state. Hence, we now assessed the effect of temperature on the folding of the MrpR wild-type protein and the MrpR^G136E^ variant *in vitro*. For this purpose, the MrpR wild-type protein and the MrpR^G136E^ variant carrying a C-terminal His_6_-tag were produced in *E. coli* and purified by Ni-NTA affinity purification. During the initial purification of MrpR, co-purification of substantial amounts of DNA was observed, indicating that MrpR is a DNA-binding protein (Supplementary Figure S1). To obtain a DNA-free MrpR protein sample an additional purification step using a Heparin column and a salt-gradient elution was performed after Ni-NTA purification (Supplementary Figure S2A, B). The absence of DNA was assessed by analyzing the molecular weight of purified MrpR by analytical size-exclusion chromatography (Supplementary Figure S2A-C and G). Next, the DNA-free proteins were analyzed for their unfolding behavior in response to increasing temperature by Nano differential scanning fluorimetry (nanoDSF). In brief, nanoDSF follows the intrinsic fluorescence of tryptophan and tyrosine residues in a protein. Consequently, the melting temperature of a protein can be calculated by monitoring the fluorescence changes when a temperature gradient is applied. While the MrpR wild-type protein exhibited an unfolding temperature of 47.8°C, the heat-sensitive MrpR^G136E^ variant unfolded approximately 10°C earlier, at a temperature of 38.4°C (Figure 3B). To assess the correct folding of MrpR^G136E^, the variant was also analyzed by analytical size-exclusion chromatography (SEC). The wild-type protein as well as the MrpR^G136E^ variant showed one symmetrical peak corresponding to the size of a MrpR monomer [calculated size: 36 kDa, experimentally determined size: 43.06 kDa for MrpR-WT and 53.8 kDa for MrpR^G136E^ (Supplementary Figure S2A, C)]. Additionally, examination of the proteins through SDS-PAGE revealed signs of degradation for MrpR^G136E^ (Supplementary Figure S2H). Hence, we assume that the G136E exchange in MrpR affects the temperature-dependent folding of the protein *in vitro*.

### Structural analysis of MrpR reveals protein fold typical for tyrosine recombinases

**Figure 4.**
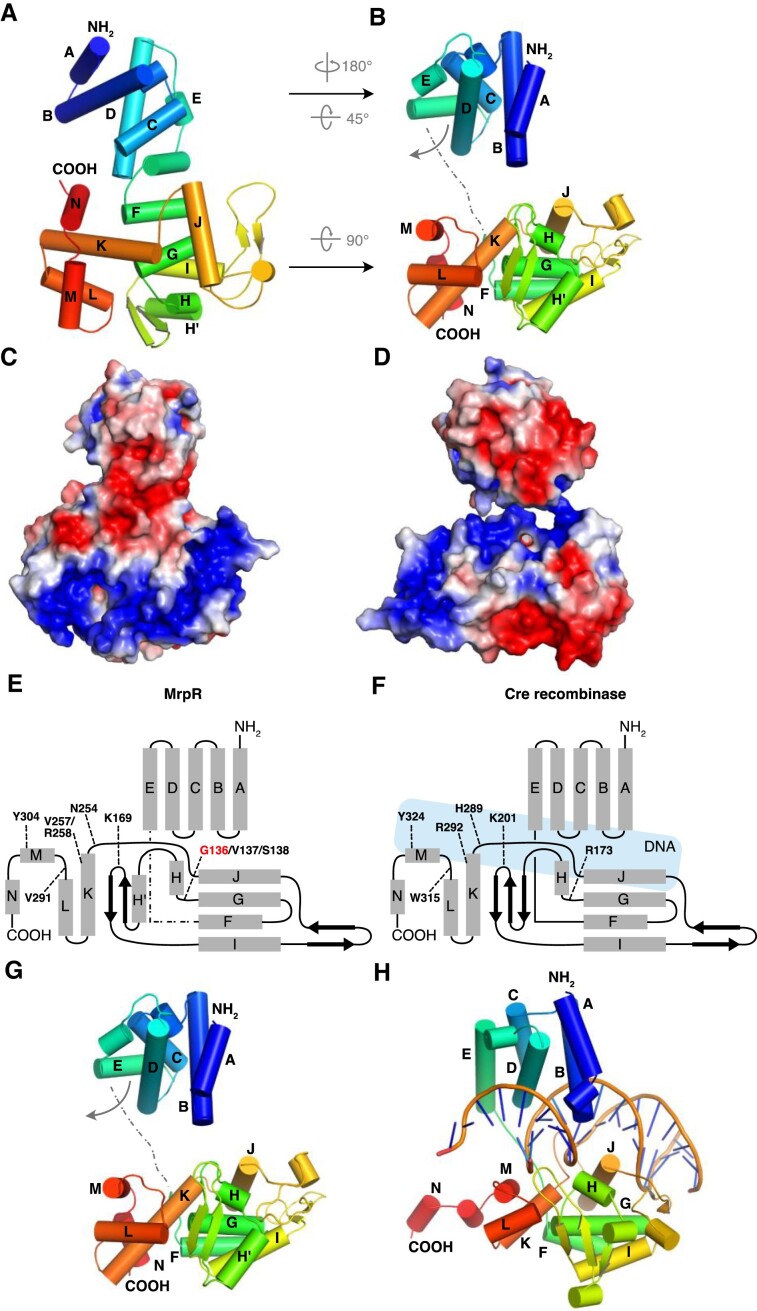
Structural analysis of the MrpR wild-type protein. (**A**) Overall fold of MrpR and representation of the 15 alpha-helices and 4 β-strands. (**B**) Structural rearrangements of the N- and C-terminal domains of MrpR required for potential DNA binding and formation of a negatively charged groove. (C, D) Electrostatic surface potential of MrpR as present in the experimentally determined crystal structure (**C**) and after the proposed structural rearrangements required for DNA binding (**D**). MrpR is colored by electrostatic surface potential, as calculated by APBS using the Pymol plug in. The color scale is the same for all proteins, ranging from −3 to +3 kT/e, with negative charges in red and positive charges in blue. (E, F) Schematic overview of MrpR (**E**) topology compared to the topology of the well-studied tyrosine recombinase and close structural homolog Cre (**F**). Residues crucial for enzymatic function of tyrosine recombinases are labeled. Location of DNA in Cre is indicated in light blue. (**G, H**) Comparison of the overall fold of MrpR (G) to Cre in its DNA bound state (PDB 1Q3U) (64).

In accordance with our observation that MrpR is a DNA binding protein, bioinformatic analysis using the amino acid sequence of MrpR identified a potential DNA breaking-re-joining catalytic domain (InterProScan5), and BLAST analysis showed weak similarities to potential integrases and recombinases. Hence, to gain molecular insight into the mode of action of MrpR, we performed a crystallographic analysis. After overproduction of MrpR fused to a C-terminal His_6_-tag in *E. coli*, the protein was purified using Ni-NTA-affinity and size-exclusion chromatography, concentrated and used for crystallization screens. Crystals formed within one week of incubation, and we determined the structure of MrpR at a resolution of 2.9 Å by molecular replacement using the N-terminal domain (residues 1–105) of the MrpR Alphafold model (51) and manually building the C-terminal part (residues 106–320) (Table 2). Two MrpR molecules were present in the crystallographic asymmetric unit (Supplementary Figure S3). The monomers interact only weakly via residues of mainly two α-helices (helix H’ and I) (Supplementary Figure S3D-F). As MrpR elutes as a monomer in analytical SEC experiments (Supplementary Figure S2A), the formation of a potential MrpR dimer appears to be an artifact of crystal packing (Figure 4, and Supplementary Figure S3). The MrpR monomer consists of 15 α-helices and 4 β-strands (Figure 4A, B; PDB ID: 8A0A). The overall structure of an MrpR monomer shows a high similarity to bacterial and phage tyrosine recombinases (Figure 4E-F and Supplementary Figure S4) (61,62). As other proteins with a tyrosine recombinase fold, MrpR harbors a CB (core-binding) domain (Gly1-Lys84, helix A–D) at the N-terminus, a catalytic (CAT) domain at the C-terminus (Tyr108-Ala320, helix F–M), which are connected by a central unfolded linker (Gly85-Leu107, helix/loop E) (Figure 4). While amino acid sequence alignments of MrpR to tyrosine recombinases do not yield a high percentage of sequence identity (below 25 percent), structural comparison using PDBefold identifies the highest similarity to the structurally and functionally characterized tyrosine recombinases Cre from *E. coli* phage P1 (63,64), XerH from *Helicobacter pylori* (65), and the integrase (Int) from *E. coli* phage Lambda (66–68) (Figure 4E-H and Supplementary Figure S4). Most of these tyrosine recombinases have been crystallized in complex with DNA to elucidate the recombination mechanisms in detail, while MrpR was crystallized in its apo form. When MrpR is structurally aligned to these proteins, the RMSD values for the CB (N-terminal domain) and CAT (C-terminal domain) range between 4 and 6 Å (Table 3). Overlay of MrpR with structures of Cre (PDB-ID: 1Q3U) (64), XerH (PDB-ID: 5JK0) (65) and Lambda Int (PDB-ID: 1P7D) (68) bound to DNA, revealed the requirement of immense structural rearrangements of MrpR during potential DNA binding. While the CB domain in the MrpR crystal structure (in absence of DNA) is rotated by approximately 180° vertically and 45° horizontally compared to Cre, XerH and Lambda Int, the CAT domain needs to be flipped by 90° horizontally (Figure 4B, E–H, and Supplementary Figure S4). We hypothesize that the observed organization of MrpR might be a crystallographic artifact since MrpR was not co-crystallized with DNA. The flexibility for the proposed rearrangements is provided by the approximately 22 residue-long linker present between the CB and CAT domain (labeled as helix E’ located between α-helices E and F), which is also present in Cre, XerH and Lambda Int (Figure 4 and Supplementary Figure S4). This structural rearrangement would also generate a positively charged groove between the CB and CAT domains of MrpR suitable for DNA interaction (Figure 4C,D). To gain structural insight into the impact of the G136E mutation within the heat-sensitive MrpR variant, we performed *in silico* mutagenesis using PyMOL. Mutation of G136 to glutamate shows, that the large side chain of the glutamate residue requires more space between the β-sheets and the α-helix K. This consequently leads to steric clashes with either F287 (for 8 rotamers) or L175/V177 (for 7 rotamers), respectively, that are offered by PyMOL upon mutation of the G136 residue (Supplementary Figure S5).

**Table 2.** Data collection and refinement statistics for MrpR

	MrpR (PDB code: 8A0A)
**Data collection**	
Space group	*P*2_1_2_1_2_1_
Cell dimensions	
	49.55
*a, b, c* (Å)	98.87
	162.24
*a, b, g* (°)	90
	90
	90
Wavelength (Å)	0.976253
Resolution (Å)	47.45–2.902
	(3.006–2.902)
R_merge_	0.3707 (2.446)
*I/σ(I)*	8.89 (1.47)
Completeness (%)	99.88 (99.27)
Redundancy	12.4 (12.7)
*CC1/2*	0.991 (0.581)
**Refinement**	
Resolution (Å)	47.45–2.902
No. reflections	226 928 (22 388)
*R_work_/R_free_*	0.2149/0.2628
No. atoms	5182
Protein	5182
Ligands/ion	0
Water	0
*B*-factor	
Protein	67.98
R.M.S. deviations	
Bond lengths (Å)	0.004
Bond angles (°)	0.75
Ramachandran	
favored (%)	97.33
allowed (%)	2.67
outliers (%)	0.00

Statistics for the highest-resolution shell are shown in parentheses.

Data were collected on ID30B (ESRF).

**Table 3.** Comparison crucial residues for the activity of tyrosine recombinases to those found in MrpR

MrpR	Cre	XerH	Lambda Int
PDB code: 8A0A	PDB code: 1Q3U	PDB code: 5JK0	PDB code: 1P7D
RMSD (Å) N-/C-terminus	4.61/4.53	6.04/5.14	4.03/4.06
V137/S138	R173	R213	R212
E144	E176	E216	D215
K169	K201	K239	K235
N254	H289	H309	H308
V257/R258	R292	R312	R311
V291	W315	H335	H333
Y304	Y324	Y344	Y342
References	(61,64,90)	(65)	(67,68,91)

### HDX-MS reveals perturbations of MrpR^G136E^ higher-order structure

The difference in melting temperature suggested to us that MrpR^G136E^ may differ in its conformation from MrpR causative for the higher instability of the former. We thus employed hydrogen/deuterium exchange mass spectrometry (HDX-MS) to ascertain potential differences in conformation between both MrpR and MrpR^G136E^. HDX-MS probes the rate with which the amide protons contained in the peptide bond exchange for protons from the solvent, the degree of which is an intrinsic characteristic of an amino acid sequence, whether the amide protons establish hydrogen bonding interactions and their solvent accessibility. Incubation of a protein in a D_2_O-containing solvent visualizes this exchange rate because a successful exchange results in a mass shift that can be quantitated by MS. Hence, HDX-MS may provide insights into a protein's higher-order structure and conformational dynamics (69). The comparison of the HDX of MrpR and MrpR^G136E^ reveals that the latter incorporates more deuterium in many parts of the protein (Supplementary Figures S6,7). These regions are mainly located in the CAT domain, but less extensive changes are also apparent in the CB domain (e.g. residues 51–63, Supplementary Figure S6B). While the G136E site of variation cannot be directly compared in this way because different peptides are obtained for either protein, areas in spatial proximity of the site of variation similarly show elevated HDX for MrpR^G136E^ (residues 250–258, Supplementary Figure S6B). Illustration of all the MrpR areas in which MrpR^G136E^ incorporates more deuterium on its crystal structure highlights that almost the entire CAT domain and the proximal portions of the CB domain (Supplementary Figure S6C) are affected. As the HDX rate is inversely correlated with the degree of higher-order structure, the higher HDX of MrpR^G136E^ than native MrpR is indicative of a less ordered conformation in most parts of this protein variant coinciding with its lower melting temperature (Figure 3B).

### Mutational and functional analysis of MrpR

**Figure 5.**
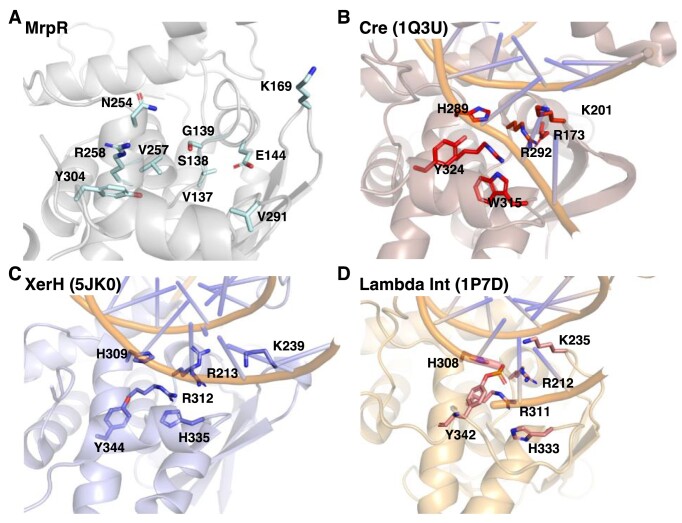
Detailed structural comparison of MrpR and the core catalytic residues of tyrosine recombinases. (**A**) Zoom into the potential DNA binding region and catalytic site of MrpR. Residues that were compared to crucial catalytic residues of tyrosine recombinases in Table 3 are shown as light blue sticks and labeled. (B–D) Comparison of the core catalytic residues in the tyrosine recombinases Cre (PDB 1Q3U; (64) (**B**), XerH (PDB 5JK0; (65) (**C**) and Lambda Int (PDB 1P7D; (68) (**D**). The catalytic residues are shown as red, blue and pink sticks, respectively. Detailed overlay of each residue with the corresponding amino acid residue found in the structure of MrpR is shown in Supplementary Figure S8.

Since our crystallographic analysis demonstrated that MrpR shares its overall fold with tyrosine recombinases, we compared the protein on the structural and functional level to already characterized proteins of the same family to gain further insights into the function of MrpR. Tyrosine recombinases are site-specific DNA modifying enzymes that bind, cleave, strand exchange and rejoin DNA at their respective, typically palindromic, recognition target sites. The function of this enzyme family has been best studied in detail for the Cre, Xer and Lambda Int recombinases. For each of these recombinases, detailed molecular studies have led to the identification of residues that are crucial for DNA recombination activity (62,64,65,68,70). The analyzed residues listed in Table 3 resemble a core active site of tyrosine recombinases, with highly conserved catalytic core: R, D/E, K, H, R, H/W and Y. These residues engage the scissile phosphate and a tyrosine nucleophile that forms a 3′ phosphotyrosine linkage with the cleaved DNA. When exemplarily compared to the closest structural homologs Cre, XerH and Lambda Int, only three of the seven residues crucial for catalysis are present in MrpR (Table 3, Figures 4E, F, 5, and Supplementary Figure S8). Especially the crucial arginine and histidine residues are substituted by serine, valine and asparagine in MrpR (Table 3, Figures 4E, F, 5, Supplementary Figure S8). Hence, it is questionable if MrpR also functions as a DNA-cleaving recombinase or rather has lost this enzymatic activity.

It has previously been shown that replacement of the nucleophilic tyrosine and the conserved lysine in the tyrosine recombinases Cre, XerH and Lambda Int by either a phenylalanine or alanine residue, respectively, render the recombinases inactive (62,64,65,68,70). Since both residues (K169 and Y304 in MrpR) are conserved in MrpR, we wanted to investigate if these residues are important for DNA binding and MrpR function. Hence, we introduced point mutations in the *mrpR* gene leading to a replacement of K169 by alanine and Y304 by alanine or phenylalanine. Each of these proteins could be produced heterologously in *E. coli* and purified (Supplementary Figure S2H). We first characterized these MrpR variants biochemically and analyzed their folding and oligomeric state via analytical size exclusion. All tested variants except MrpR^Y304A^ showed a monomeric protein peak in this analysis (Supplementary Figure S2D–G). MrpR^Y304A^ possessed a peak shoulder, showed bands of degradation in SDS-PAGE analysis and large parts of the protein were insoluble during overproduction indicative of problems in protein folding and stability (Supplementary Figure S2E, H). We further tested the thermostability of these variants using nanoDSF. While the MrpR^K169A^ variant showed the same thermal unfolding behavior as the wild-type MrpR protein, the thermal stabilities of the MrpR^Y304F^ and MrpR^Y304A^ variants were reduced by about 6°C (Figure 3B) comparable to the initially tested heat-sensitive MrpR^G136E^ variant. This inference is also substantiated by changes in HDX-MS of MrpR^Y304F^ compared to native MrpR, which are highly reminiscent in quality and quantity to those observed for the heat-sensitive MrpR^G136E^ variant (Supplementary Figures S6 and S7).

### MrpR does not show recombination activity

Since MrpR possesses a similar fold as tyrosine recombinases and at least harbors the catalytically important tyrosine and lysine residue, we wondered if MrpR might function as a recombinase *in vitro* using the *attL* and *attR* sites of the SPβ phage (14). To assess the activity of MrpR WT, and the MrpR^G136E^, MrpR^Y304F^ and MrpR^K169A^, we employed an *in vitro* recombination assay previously described by Abe et al. (12) with minor changes as we used fluorescently labeled fragments of the *attL* (242 bp, labeled with Cy5) and *attR* (616 bp, labeled with Cy3). If MrpR would be able to cleave, strand exchange and rejoin the SPβ recombination sites, we would expect the appearance of a hybrid DNA fragment with the size of 426 bp carrying both fluorescence labels (Supplementary Figure S9A). MrpR wild-type and the tested MrpR^G136E^, MrpR^Y304F^ and MrpR^K169A^ variants were incubated with the labeled *attL* and *attR* sites for 60 min at 37°C, boiled to release MrpR from the DNA and analyzed on a native polyacrylamide gel. Neither MrpR wild-type nor any of the tested MrpR variants resulted in the formation of a hybrid DNA fragment carrying both fluorescence labels, which would be indicative of a DNA recombination event of the SPβ *att* sites (Supplementary Figure S9B).

Next, we assessed the *in vivo* activities of the MrpR^K169A^, MrpR^Y304F^ and MrpR^Y304A^ variants by performing a heat shock experiment. For this purpose, we introduced the *mrpR*^A911T^ (Y304F), *mrpR*^TAT910-912GCG^ (Y304A) and *mrpR*^AAG505-507GCG^ (K169A) alleles into the *amyE* locus of the strain KK002 (SPβ c2). Next, we deleted the native *mrpR*^G407A^ copy from the SPβ c2 genome resulting in the strains KK120 (SPβ c2 [*mrpR*^G407A^*::ermC*] *P_alf4_-mrpR*^A911T^), KK121 (SPβ c2 [*mrpR*^G407A^*::ermC*] *P_alf4_-mrpR*^TAT910-912GCG^) and KK138 (SPβ c2 [*mrpR*^G407A^*::ermC*] *P_alf4_-mrpR*^AAG505-507GCG^). The fact that it was possible to delete the native *mrpR*^G407A^ allele indicates that the MrpR^K169A^, MrpR^Y304F^ and MrpR^Y304A^ variants were functional because they prevented the entry of the SPβ *mrpR* mutant to enter the lytic cycle under unstressed conditions (Figure 3C). Consistent with the nanoDSF experiments (Figure 3B), only the MrpR^Y304F^ and MrpR^Y304A^ variants but not MrpR^K169A^ showed increased temperature sensitivities (Figure 3C). Thus, the residues G136 and Y304 are critical for the thermal stability of MrpR and potential DNA interaction. Moreover, it is unlikely that MrpR functions as a recombinase because the replacement of the tyrosine residue, which is crucial for enzyme catalysis of known recombinases (see above), still allows the phage to enter the lytic cycle and form infectious particles.

### Genome-wide profiling of MrpR binding by ChAP-seq

**Figure 6.**
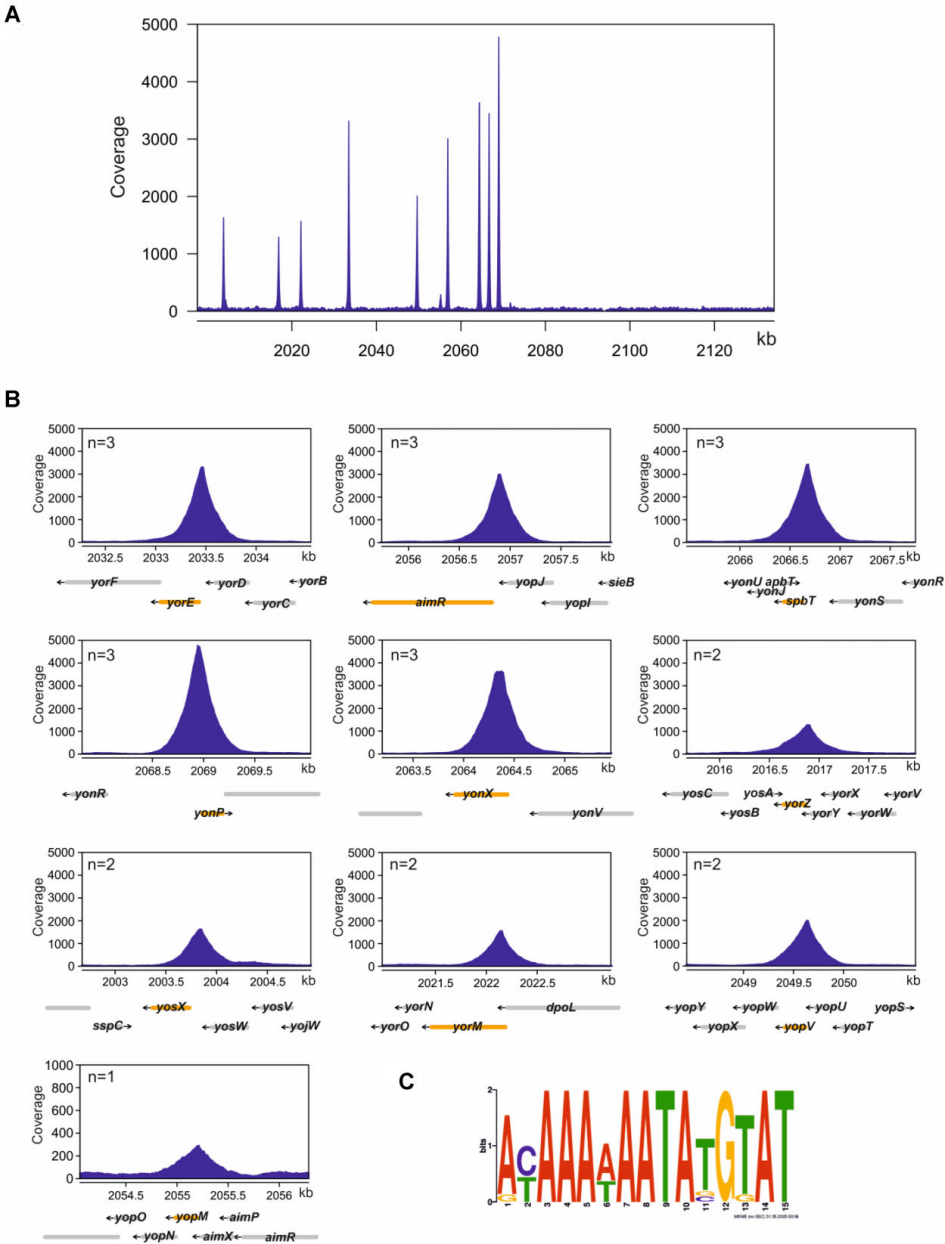
Genomic targets bound by MrpR. (**A**) Mapping of ChAP-seq reads for MrpR-Strep binding to the prophage region of the *B. subtilis* genome. The coverage of binding peaks (y-axis) was plotted against the respective genomic regions of *B. subtilis* (x-axis). (**B**) Excerpts from peaks for chosen prophage targets. Genes are indicated below with grey arrows, while orange arrows represent the respective gene the peak is ascribed to. The number of replicates where a significant peak could be found is shown in each respective graph as ‘*n*’. (**C**) Sequences of the 9 peaks were used to derive a DNA binding motif using MEME-ChIP (89). A 15 bp-long motif was identified (*E*-value = 3.9 × 10^−20^).

Given that MrpR does not function as a recombinase, but significant amounts of DNA were isolated during its purification, MrpR might have evolved to a DNA-binding protein with solely regulatory function. Therefore, we determined the genome-wide binding profile of MrpR by combining affinity chromatography purification of cross-linked MrpR-DNA complexes with sequencing of associated DNA (ChAP-Seq) as described previously (56,57). Prior to the ChAP-Seq analyses, we assessed whether the Strep-tag affects the *in vivo* activity of MrpR. For this purpose, we took advantage of the dominance of the wild-type variant over the mutant variant, i.e. the fact that heat inducibility is lost when a wild-type copy of *mrpR* is present in the strain and introduced the *mrpR-Strep* allele into the *amyE* locus of the *B. subtilis* strain KK002. Next, we replaced the native *mrpR^G407A^* gene with an erythromycin resistance gene by transformation (KK152). The heat shock experiment revealed that the Strep-tag did not affect the *in vivo* activity of MrpR. The following ChAP-Seq experiments with the MrpR-Strep protein allowed us to detect ten prominent peaks, which are all located inside the SPβ genome (Figure 6A and Supplementary Figure S10A). In contrast, no peaks could be detected with the control strain KK167 synthesizing the untagged MrpR^G136E^ protein (Supplementary Figure S10B). A closer inspection revealed that the peaks are in the promoter regions of the *yorE, aimR, spbT* (*yonT*), *yonP, yonX, yorZ, yosX, yorM, yopV* and *yopM* genes that have been previously identified to contain the SPBRE (SPbeta repeated element) (31) (Figure 6B). Except for the *aimR, spbT* and *yopM* genes, the functions of the remaining genes are currently unknown. As described above, *aimR* codes for the transcriptional regulator AimR that in its apo form activates transcription of the AimX ncRNA that promotes the lytic cycle (Figure 1). The *spbT* gene codes for a toxin and needs to be silenced by the RNase III of the host (71). *yopM* is part of the *yopMNOPQR* operon and encodes the YopM protein that suppresses lytic propagation of the phage population via abortive infection by binding to the MazE antitoxin of the type MazF/MazE toxin-antitoxin (TA) complex (72). Thus, MrpR likely controls the expression of a component of the arbitrium system and either directly or indirectly modulates the activity of TA systems. To deduce the binding motif of MrpR, the sequences of all identified peaks were extracted and analyzed using the MEME-ChIP platform (73). A 15-bp long motif was predicted that exactly matches the SPBRE (31) (Figure 6C). To conclude, the ChAP-Seq analyses indicate that the SPBRE is the recognition site for MrpR, the master repressor of the lytic cycle of SPβ.

### *In vitro* DNA-binding activity of MrpR

**Figure 7.**
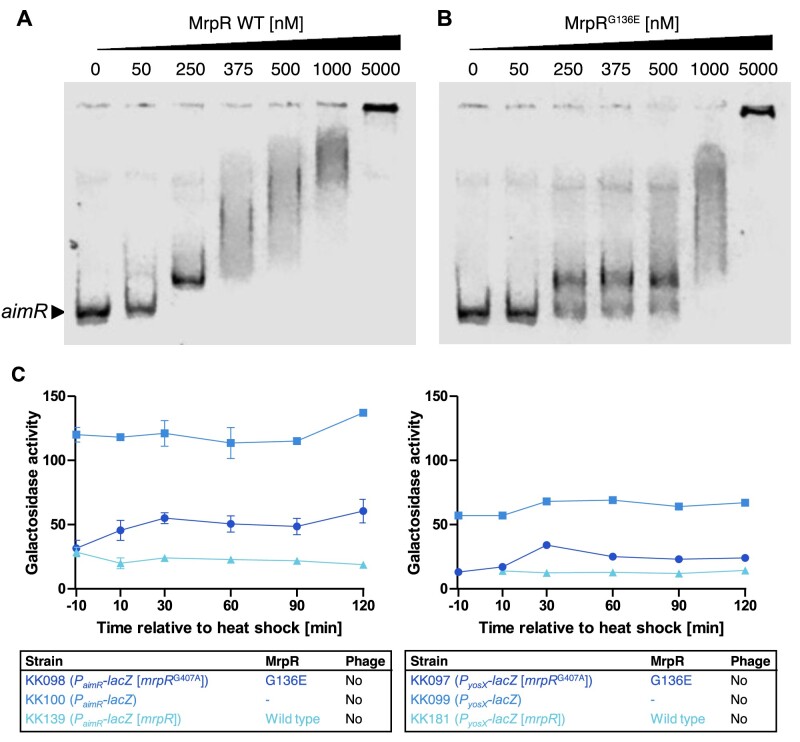
*In vitro* and *in vivo* analysis of MrpR DNA binding. (A, B) Electrophoretic mobility shift assays (EMSAs) showing the DNA-binding activities of the MrpR wild-type protein and the MrpR^G136E^ variant. (**A**) Complex formation between the MrpR wild-type protein and the *P_aimR_* DNA fragment carrying the SPBRE site. (**B**) Complex formation between the MrpR^G136E^ variant and the *P_aimR_* DNA fragments carrying the SPBRE site. See Materials and Methods for the experimental procedure. (**C**) β-Galactosidase activity assays to assess the DNA-binding activity of wild-type MrpR and MrpR^G136E^. The indicated strains were grown in LB medium for 2.5 h at 37°C, transferred for 10 min to 50°C, and further cultivated for 2.5 h at 37°C. The β-galactosidase activity is given as units per milligram protein. Experiments were carried out at least three times. Boxes indicate mean values.

Next, we assessed binding of MrpR to a DNA fragment carrying the SPBRE *in vitro*. Hence, electrophoretic mobility shift assays (EMSAs) using purified DNA-free MrpR and MrpR^G136E^ were performed with the fragments upstream of *aimR* harboring the SPBRE and the *attR* region as a control. While the MrpR wild-type protein only required the presence of 5-fold protein excess to result in a shift of the DNA fragment, the MrpR^G136E^ variant showed a decrease in binding affinity (approx. 10- to 20-fold excess of MrpR^G136E^ required for a comparable DNA shift) (Figure 7A, B). Since we observed co-purification of DNA during heterologous production and purification of MrpR in *E. coli* (Supplementary Figure S1), we also tested *in vitro* binding of MrpR to a *B. subtilis* DNA fragment lacking the SPBRE. Hence, we employed the *attR* region at the corner of the SPβ genome in an EMSA. This resulting EMSA also indicates a weak DNA binding behavior of the wild-type MrpR and MrpR^G136E^ variant protein to the SPBRE-less DNA region (Supplementary Figure S11). While for MrpR wild-type a shift of the DNA–protein complex using the *attR* region was observed upon addition of approximately 10- to 20-fold protein excess, a shift of the *attR* region was only observed in a DNA:protein-ratio of 1:100 with the MrpR^G136E^ variant (Supplementary Figure S11). Hence, we hypothesize, that additional factors might be required for the specificity of MrpR SPBRE-DNA binding *in vivo*.

To further assess the reduced binding affinity, we performed binding studies using isothermal titration calorimetry (ITC). We employed the region upstream of the *aimR* promoter and added increasing amounts of the MrpR wild-type protein or the MrpR^G136E^ variant. While the wild-type protein had a DNA binding affinity of 0.238 ± 0.08 μM (Supplementary Figure S12A, C), the binding affinity of the MrpR^G136E^ variant was decreased by approximately 7-fold to 1.69 ± 0.69 μM (Supplementary Figure S12B, D). When we then assessed the previously described MrpR^K169A^ and MrpR^Y304F^ variants for their *in vitro* DNA binding affinity via ITC using the *aimR* region, we observed that the MrpR^K169A^ variant possessed a DNA binding affinity in a similar range as MrpR wild-type in the ITC analysis (0.238 ± 0.08 μM for MrpR wild-type and 0.346 ± 0.15 μM for MrpR^K169A^), while the Y304F exchange resulted in a phenotype comparable to the heat sensitive G136E mutant (1.69 ± 0.69 μM for MrpR^G136E^ and 1.34 ± 0.5 μM for MrpR^Y304F^) (Supplementary Figure S12). These *in vitro* results complete the picture from the *in vivo* heat-shock experiment, in which a strain expressing MrpR^Y304F^ showed a heat-sensitive phenotype like a strain encoding MrpR^G136E^, while a strain carrying MrpR^K169A^ behaved like the wild-type (Figure 3C). To further consolidate the changes in DNA binding, we performed HDX-MS on MrpR, MrpR^G136E^ and MrpR^Y304F^ in the presence of DNA and compared the HDX rate of those to their respective apo states (Supplementary Figures S7 and S13). The protein regions exhibiting reduced HDX in the presence of DNA are similar for all three variants except for the linker region between helices E and F and the C-terminal MrpR portion encompassing helices K to N (Supplementary Figure S13). These concur with the areas where MrpR^G136E^ and MrpR^Y304F^ showed the most pronounced defects in higher-order structure (Supplementary Figure S6). Collectively, these data suggest that both variants are, in principle, still capable of DNA coordination via the CAT domains’ N-terminal portion and that their reduced DNA binding affinity is causally linked to structural defects in the C-terminal part of the CAT domain (Supplementary Figure S13C). To conclude, the *in vitro* analyses confirm that MrpR binds DNA fragments carrying the SPBRE with high affinity and that the affinity is reduced in the MrpR^G136E^ and MrpR^Y304F^ variants.

### Regulation of *P_aimR_* and *P_yosX_* promoters by MrpR

Following the *in vitro* analysis, we assessed the effect of the environmental temperature on the *in vivo* DNA-binding activity of MrpR and MrpR^G136E^. For this purpose, we constructed translational *P_aimR_-lacZ* and *P_yosX_-lacZ* fusions that contain the SPBRE and the promoters (31) and integrated them into the *amyE* locus of the *B. subtilis* genome. The strains were cultivated for 2.5 h at 37°C, heat shocked for 10 min at 50°C, and further incubated for 2 h at 37°C. The activity of the β-galactosidase was measured 10 min before the heat shock and 10, 30, 60, 90 and 120 min after the heat shock (Figure 7C). As shown in Figure 7C, prior to the heat shock, the activity of both promoters was five-fold reduced when either MrpR or MrpR^G136E^ was present. After the heat shock, the activity of the *P_aimR_* and *P_yosX_* promoters slightly increased in the strains synthesizing the MrpR^G136E^ variant. By contrast, the activity of the promoters remained low in the strain producing the MrpR wild-type protein. Thus, MrpR and MrpR^G136E^ negatively control the activity of the *P_aimR_* and *P_yosX_* promoters *in vivo* and MrpR^G136E^ responds to the ambient temperature.

### Heat induction of the lytic cycle of SPβ c2 does not depend on SprB

**Figure 8.**
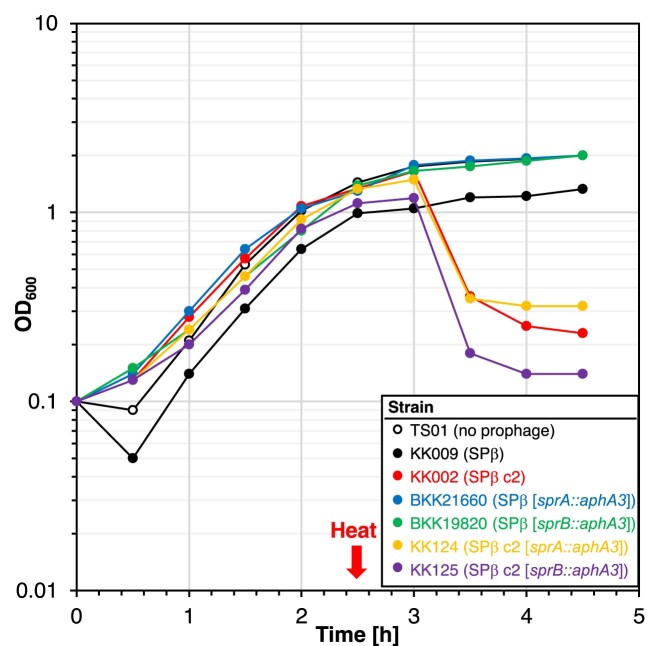
Heat-induced cell lysis of *B. subtilis* carrying SPβ c2 does not require SprA or SprB. The indicated strains were grown for 2.5 h at 30°C, heat shocked at 50°C for 10 min and further cultivated at 30°C. The square brackets indicate the *sprA* and *sprB* deletions in the phage genome. The OD_600_ was monitored over time. Single points from one representative of three replicates are shown.

Previously, it was shown that SprA and SprB are required for SPβ prophage excision during sporulation (Figure 1) (12). We were wondering if the heat-inducible SPβ excision of the c2 mutant also depends on SprA and SprB, especially since *sprB* is under the control of a sporulation-dependent promoter (14). To test this idea, we deleted the *sprA* and *sprB* genes in the strain KK002 carrying SPβ c2. Genome sequencing verified the deletion of the *sprA* and *sprB* genes in the strains KK124 (*sprA::aphA3*) and KK125 (*sprB::aphA3*), respectively. Both strains also carried the four mutations that were identified in the SPβ c2 phage (Table 1). Next, we performed a heat shock experiment and assessed cell lysis of the strains KK124 (SPβ c2 [*sprA::aphA3*]) and KK125 (SPβ c2 [*sprB::aphA3*]). As shown in Figure 8, both strains are still lysed in the absence of either SprA or SprB. However, only the *sprB* mutant produced infectious SPβ c2 phage particles as verified by plaque assays using the prophage-free strain TS01 (data not shown). We conclude that the heat-inducible SPβ excision of the c2 mutant also depends on the serine recombinase SprA, while the heat-inducible induction of lysis is independent of SprA and SprB.

### Identification of YosL, a novel component of the lysis-lysogeny management system of SPβ

**Figure 9.**
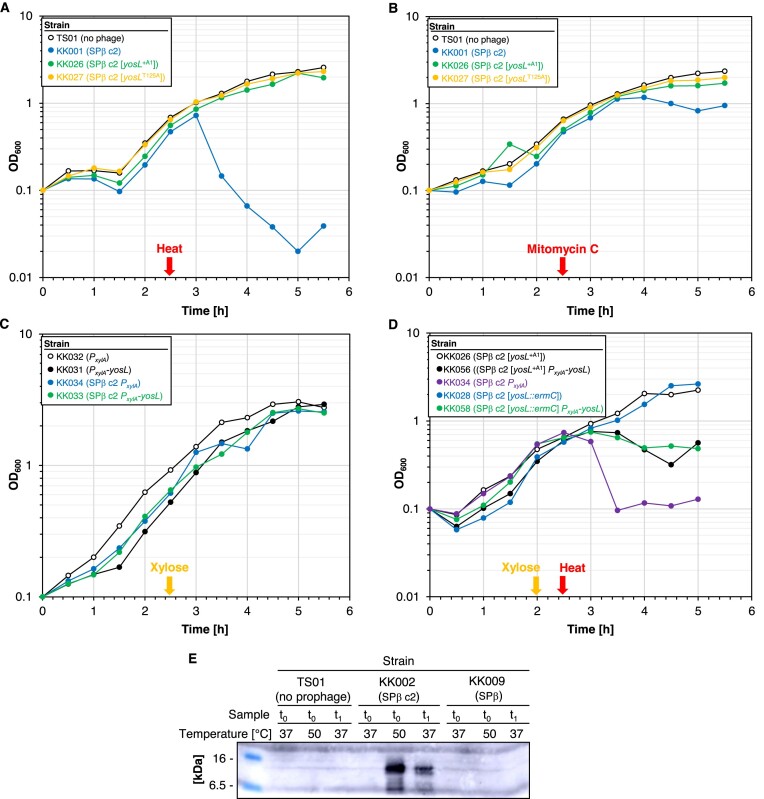
YosL, a novel player in the lysis/lysogeny management system of SPβ. (**A**) Characterization of evolved SPβ c2 suppressor mutants. All strains were grown for 2.5 h at 37°C, heat shocked at 50°C for 10 min and further cultivated at 37°C. The OD_600_ was monitored over time. (**B**) Effect of mitomycin C on prophage induction in strains carrying SPβ c2 and its suppressors during growth in LB at 37°C. Mitomycin was added to a final concentration of 0.5 μg ml^−1^ and OD_600_ was monitored over time. (**C**) Effect of xylose (0.5% (w/v)) on prophage induction in the indicated strains that were grown in LB. (**D**) Effect of xylose (0.5% (w/v)) and heat (10 min at 50°C) on prophage induction in the indicated strains that were grown in LB. Single points from one representative of three replicates are shown. (**E**) Cellular amounts of YosL were determined by western blotting. Crude extracts of the indicated strains were isolated from exponentially growing LB cultures at timepoints *t*_0 37°C_ (before the heat shock), *t*_0_50°C (immediately after the heat shock) and *t*_1_37°C(30 min after the heat shock). After electrophoresis in a 15% SDS-PAGE and transfer onto a PVDF membrane, YosL was detected by rabbit polyclonal antibodies raised against YosL-His_6_. 20 mg of crude extracts were applied.

When performing heat shock experiments with the strain KK001 carrying the SPβ c2 prophage, we observed that approximately 10% of the cells survived. Characterization of individual isolates of the surviving cells revealed that the lytic cycle of SPβ c2 was still inducible by heat, indicating the presence of intact prophages (data not shown). We speculated that among the surviving cells, there could be SPβ c2 mutant variants that had acquired intra- or extragenic mutations, which would prevent heat inducibility of the lysogens. To isolate SPβ c2 mutant variants that had lost the heat-sensitive phenotype, we selected suppressor mutants. For this purpose, the strain KK001 (SPβ c2) was grown at 37°C until the culture reached an OD_600_ of about 0.8, heat shocked for 10 min at 50°C and incubated for additional 6 h at 37°C. After 2 h of incubation cell lysis took place, which could be recognized by the decrease in optical density. When the culture again reached an OD_600_ of 0.8, fresh medium was inoculated to an OD_600_ of 0.1 and incubated overnight at 37°C. Next day, cells from the overnight culture were used to inoculate fresh medium to an OD_600_ of 0.1, and the heat shock cycle was repeated. After performing this cycle for 7 days, cell lysis could no longer be induced by heat, suggesting that genes of the SPβ lysis-lysogeny management system had probably acquired detrimental mutations. Next, 15 clones from the evolved culture were isolated, and the presence of the prophage was verified by a sublancin assay. The culture supernatants of five randomly selected clones were used to generate lysogens in the background of the prophage-free strain TS01. In two of the 19 isolated prophage-containing strains that were designated as KK026 and KK027, cell lysis could no longer be induced by heat (Figure 9A). Genome and subsequent Sanger sequencing revealed that the SPβ c2 prophage had acquired mutations in the *yosL* gene in both strains. In strain KK026, a single-nucleotide insertion (+A1) would lead to a frameshift that truncates the YosL protein after only four of the predicted 117 amino acids. The strain KK027 had a single-nucleotide exchange in *yosL* (T125A) that likewise would truncate YosL after 59 amino acids. We also observed that cell lysis of the strains KK026 (SPβ c2 [*yosL*^+A1^]) and KK027 (SPβ c2 [*yosL*^T125A^]) could not be induced by treatment with mitomycin C (Figure 9B). Thus, YosL is essential for the heat- and mitomycin C-dependent induction of the lytic cycle of SPβ c2.

Since the *yosL* gene is not expressed in the dormant state of the SPβ prophage (26), *yosL* transcription must be activated when the phage enters the lytic cycle in an MrpR-dependent manner. However, to assess whether YosL can trigger the lytic cycle independent of MrpR^G136E^, we constructed the plasmid pRH168 allowing the xylose-dependent expression of the *yosL* gene from the *ganA* locus. YosL is not toxic for the bacteria because the prophage-free control strains KK031 (*P_xylA_-yosL*) and KK032 (*P*_xylA_, control) grew in the presence of xylose (Figure 9C). Moreover, the expression of *yosL* in the strain KK033 (SPβ c2 *P_xylA_-yosL*) carrying the prophage did not result in cell lysis (Figure 9C). Next, we tested whether the artificially expressed *yosL* gene complements the spontaneously inactivated *yosL*^+A1^ allele and the deletion of *yosL* in the strains KK056 (SPβ c2 [*yosL*^+A1^] *P_xylA_-yosL*) and KK058 (SPβ c2 [*yosL::ermC*] *P_xylA_-yosL*), respectively, upon heat shock. The parental strains KK028 (SPβ c2 [*yosL::ermC*]) and KK026 (SPβ c2 [*yosL*^+A1^]) served as controls. As shown in Figure 9D, albeit to a lesser extent compared to the control strain KK034 (SPβ c2 *P_xylA_*), the xylose-dependent expression of *yosL* in the strains KK056 (SPβ c2 [*yosL*^+A1^] *P_xylA_-yosL*) and KK058 (SPβ c2 [*yosL::ermC*] *P_xylA_-yosL*) induced lysis after heat treatment. Thus, YosL depends on MrpR^G136E^ to activate the lytic cycle of SPβ.

To assess whether the heat shock affects the synthesis of the YosL protein in the strain KK002 (SPβ c2), we performed a Western blot using polyclonal antibodies raised against YosL. The prophage-free strain TS01 and the strain KK009 (SPβ) served as controls. Samples for the preparation of cell-free crude extracts were taken during cultivation at 37°C before and 30 min after applying a heat shock at 50°C. As expected, YosL was not synthesized in the strains TS01 (no prophage) and KK009 (SPβ) (Figure 9E). By contrast, immediately after the heat shock, the strain KK002 (SPβ c2) synthesized a protein corresponding to YosL (13 kDa). Hence, we identified YosL as a novel component of the lysis-lysogeny management system that is required for the heat- and mitomycin C-dependent induction of the lytic cycle of the SPβ c2 mutant.

## DISCUSSION

Previously, it has been shown that the *mrpR* gene codes for the master repressor MrpR of the lytic cycle of the SPβ prophage, which is present in the *B. subtilis* laboratory strain 168 (25,26). Here, we confirmed that MrpR is a key player in the lysis-lysogeny management system of SPβ. We show that a single mutation in the *mrpR* gene enables induction of the SPβ c2 lytic cycle by transiently increasing the cultivation temperature. This finding suggested that either the stability or the folding of the encoded MrpR^G136E^ protein is influenced by the ambient temperature of the bacterial culture. The first possibility could be excluded because the cellular levels of MrpR^G136E^ were not affected by the ambient temperature (Figure 3A). In contrast, biochemical characterization of the MrpR^G139E^ variant revealed that its unfolding temperature was decreased by about 10°C and that this variant exhibited less higher-order structure (Figure 3B and Supplementary Figures S6,7). Inspection of the MrpR structure furthermore suggests that the glycine to glutamate exchange in the heat-sensitive mutant requires more space between the β-sheets and the α-helix K and thus leads to steric clashes with either F287 or L175/V177, respectively (Figure 4E and Supplementary Figure S5). Thus, the amino acid exchange in the MrpR protein rather renders the folding of the protein sensitive to temperature and reduces its DNA binding affinity.

Our structural characterization of MrpR revealed that the monomer shows a high similarity to well-known bacterial and phage tyrosine recombinases (Table 3, Figure 4, Supplementary Figures S4 and S8). While mutational and functional analysis showed that MrpR lacks most residues crucial for enzymatic function, the conserved tyrosine residue (Y304) seems to be crucial for the DNA-binding activity of the protein (Figure 4 and Supplementary Figure S12). Yet, the precise role of Y304 must be determined by structural characterization of MrpR-DNA complexes. However, it is interesting to note that the replacement of tyrosine by phenylalanine or alanine at position 304 also renders the MrpR^Y304F^ and MrpR^Y304A^ variants temperature sensitive, albeit to a lesser extent than the G136E exchange in MrpR^G136E^ (Figure 3B and C). As similar exchanges of the catalytic tyrosine residue in tyrosine recombinases such as Cre, XerH or Lambda Int render the proteins inactive/incapable of DNA recombination (62,64,65,70), this strengthens the conclusion that MrpR does not act as a tyrosine recombinase despite the structural similarities. Instead, we assume that MrpR has fully lost recombinase function and evolved into a DNA-binding repressor protein. Thus, MrpR is structurally related to a tyrosine recombinase but functions as a regulatory protein. There are several examples of the evolution of an enzyme to a DNA-binding regulator (74). For instance, the DNA-binding repressor protein Mlc from *Thermus thermophilus* responds to glucose that binds to a motif, which is conserved in glucose kinases (75,76). In fact, integrases have been described as acting as repressors to modulate their own synthesis (77,78). Probably, the recruitment of tyrosine recombinases as DNA-binding transcription factors is more widespread than previously anticipated.

It has been suggested 24 years ago by in silico analysis that the SPBRE might represent the recognition site for an SPβ prophage repressor (31), which has now been identified as MrpR. The genome-wide profiling of MrpR binding now experimentally confirmed binding of MrpR to the SPBRE elements within the SPβ prophage. The identification of the MrpR recognition site in the *P_aimR_* promoter region and our expression analysis revealed that the repressor is involved in the regulation of the arbitrium system (Figures 6B and 7A, C). Thus, like the AimP-hexapeptide-dependent control of AimR, the MrpR-dependent regulation of *aimR* expression is crucial to prevent the killing of the entire bacterial population by SPβ, thereby ensuring long-term prophage maintenance. A recent study uncovered another link between the arbitrium system and the repressor MrpR (72). It has been shown that the AimP-hexapeptide of the arbitrium system activates the *yopMNOPQR* operon, which contains the *mrpR* gene. Interestingly, the YopM protein, which is also encoded by the *yopMNOPQ-mrpR* operon, directly binds to the MazE antitoxin of the type II MazF/MazE (TA) complex, and suppresses lytic propagation of the phage population via abortive infection (72). Thus, the arbitrium system and the MrpR regulon are linked to each other in multiple ways and a host TA module plays a key role in the arbitrium-regulated inhibition of the lytic propagation of SPβ.

We are convinced that the heat-inducible lysis phenotype of a strain encoding the MrpR^G136E^ mutant will further aid to gain a deeper understanding of the complex regulatory networks of the lysis-lysogeny decision system of SPβ and other SPβ-like phages. For example a temperature-sensitive variant of the repressor of the lysogenic phage λ was shown to be helpful to study the underlying molecular mechanisms by which DNA-binding transcription factors exert their function (79–82). Moreover, the entry into the host environment and the accompanied temperature change serve as cues for thermo-sensing transcription factors to control virulence gene expression in pathogenic bacteria (83). Temperature-sensitive DNA-binding transcription factors have also been employed for constructing expression systems that can be triggered by an ambient temperature (84,85). In case of MrpR, it is unlikely that the temperature serves as a signal for the SPβ wild-type prophage to enter the lytic cycle due to the inactivation of MrpR because a heat shock does affect its repressor function. Thus, additional research is needed to understand which factors trigger the release of MrpR from its DNA binding sites and elucidate the role of the genes regulated by MrpR.

Previously, it has been shown that SprA and SprB are key components required for the excision of the SPβ genome during sporulation (Figure 1) (12). Here, we could show that lysis of a strain carrying the SPβ c2 allele was induced when either *sprA* or *sprB* were absent (Figure 8). The fact that only the strain lacking the *sprA* gene did not produce infective phage particles indicates that the heat-inducible SPβ excision of the c2 mutant also depends on the serine recombinase SprA but probably not on SprB. However, it is not unlikely that additional factors and regulatory circuits control prophage excision during sporulation or due to mitomycin C treatment. First, SprB overproduction was shown to trigger prophage excision but not the lytic cycle (12). Second, a genome-reduced variant of SPβ containing only the *sprA* and *sprB* genes retained the ability to rearrange the *spsM* gene but did not undergo excision during mitomycin C treatment (12). It will be interesting to elucidate which yet unknown prophage- or the host-derived factors are involved in the excision of the SPβ.

As mentioned above, temperature-sensitive variants of DNA-binding proteins were helpful to study the functions of DNA-binding transcription factors. Here, we took advantage of the temperature-sensitive MrpR^G136E^ variant to identify the YosL protein as a novel component of the lysis-lysogeny management system of SPβ. We showed that the YosL protein was synthesized when a *B. subtilis* lysogen carrying the SPβ c2 prophage was exposed to heat (Figure 9). A bioinformatic analysis revealed that YosL is predicted to possess an Arc-type ribbon-helix-helix domain, which is also present in the structurally homologous repressors Mnt and Arc of the phage P22, that employs both repressors for maintaining the lysogenic state (86,87). There are two possibilities how the YosL protein might activate the SPβ c2 lytic cycle. First, elevated cellular amounts of YosL could inhibit the synthesis of another factor that prevents the synthesis of components activating the lytic cycle of SPβ c2. Second, YosL might directly activate yet unknown factors triggering the lytic cycle of SPβ c2. However, future structural and functional analysis is required to gain deeper insights into the regulatory role of YosL. Surprisingly, we found that the overexpression of YosL alone did not activate the lytic cycle of SPβ (Figure 9). A similar observation has been made when the transcription factor AimR that activates the expression of the nc RNA AimX, which in turn activates the lytic cycle of SPβ, is overexpressed (25). These findings suggest that AimR and YosL act in concert with other yet unknown components to allow the switch from the lysogenic to the lytic cycle of SPβ. Interestingly, it was recently proposed for the SPβ-like prophage phi3T, that not only AimX but also YosL are MazE-like antitoxins blocking the MazF toxin and thereby curbing host defense via this toxin-antitoxin system (88).

Finally, our present study opens a wealth of interesting questions that remain to be answered in the future. For instance, the molecular details of how the arbitrium system and the MrpR regulon influence each other need to be elucidated. Moreover, what are the factors governing the DNA-binding specificity of MrpR? Furthermore, the function of YosL and other players of the lysis/lysogeny management system, such as YopN remain to be uncovered.

## SOURCE DATA

Supplemental Dataset. The spreadsheet provides a summary of the conditions used for HDX-MS analyses and a full list of the peptides and residue-specific HDX obtained for MrpR, MrpR^G136E^ and MrpR^Y304F^.

## Supplementary Material

gkad675_Supplemental_File

## Data Availability

Coordinates and structure factors have been deposited within the protein data bank (PDB) under the accession code: 8A0A. Sequencing data for this study have been deposited in the European Nucleotide Archive (ENA) at EMBL-EBI under accession number PRJEB63141 (https://www.ebi.ac.uk/ena/browser/view/PRJEB63141). The authors declare that all other data supporting the findings of this manuscript are available within the article and its supplementary data files.
