# Supplementary material for: Structural and functional characterization of MrpR, the master repressor of the Bacillus subtilis prophage SPβ
Source: Nucleic Acids Res. 2023 Aug 21;51(17):9452–74. doi: 10.1093/nar/gkad675 (PMC10516654; doi:10.1093/nar/gkad675)
Supplement: gkad675_Supplemental_File [file gkad675_supplemental_file.docx]

*Supplementary Material for:*

**Structural and functional characterization of MrpR, the master repressor of the *Bacillus subtilis* prophage SPβ**

Katharina Kohm^1,2,7^, Ekaterina Jalomo-Khayrova^3,7^, Aileen Krüger^4^, Syamantak Basu^1^, Wieland Steinchen^5^, Gert Bange^3,5^, Julia Frunzke^4^, Robert Hertel^1,6,*^, Fabian M. Commichau^1,2,*^, Laura Czech^3,*^

^1^FG Synthetic Microbiology, Institute for Biotechnology, BTU Cottbus-Senftenberg, Senftenberg, Germany

^2^FG Molecular Microbiology, Institute for Biology, University of Hohenheim, Stuttgart, Germany

^3^Center for Synthetic Microbiology (SYNMIKRO) and Department of Chemistry, Phillips-University Marburg, Marburg, Germany

^4^Institute of Bio- and Geosciences, iBG-1: Biotechnology, FZ Jülich, Germany

^5^Max-Planck Institute for Terrestrial Microbiology, Marburg, Germany

^6^Department of Genomic and Applied Microbiology, Institute of Microbiology and Genetics, Georg-August-University of Göttingen, Göttingen, Germany

^7^Equally contributing

*Corresponding authors:

czechla@staff.uni-marburg.de; fabian.commichau@uni-hohenheim.de

Universität Hohenheim

70699 Stuttgart

Phone: +49-711459-22222

Mail: fabian.commichau@uni-hohenheim.de

Phillips-Universität Marburg

35043 Marburg

Phone: +49-177-7555527

Mail: czechla@staff.uni-marburg.de

**RUNNING TITLE:** Control of the SPβ phage lysogeny management system

**KEYWORDS:** prophage, regulation, repressor, MrpR, recombinase, suppressor mutant

**Figure S1. DNA contamination during purification of MrpR and MrpR^G136E^. A, B.** Size-exclusion chromatography (SEC) of MrpR WT (**A**) and MrpR^G136E^ (**B**). Absorption at 280 nm is shown in blue, and 254 nm in orange. **C.** Analysis of co-purified DNA present in affinity purified MrpR WT and MrpR^G136E^ using agarose gel electrophoresis. Lanes are labelled within the figure.

**Figure S2. Analytical SEC and SDS PAGE analysis of MrpR and MrpR variants. A.** Analytical SEC of MrpR WT after additional heparin purification. Comparison to standard protein solutions is shown in the smaller graph. **B.** SDS-PAGE analysis of SEC fractions of MrpR WT protein. **C-F.** Analytical SEC analysis of the variants MrpR^G136E^ (**C**), MrpR^Y304F^ (**D**), MrpR^Y304A^ (**E**) and MrpR^K169A^ (**F**). **G.** Comparison of MrpR WT and variants to SEC of standard protein solutions (thyroglobulin [660 kDa], ferritin [474 kDa], aldolase [160 kDa], conalbumin [76 kDa], ovalbumin [43 kDa] and ribonuclease A [13.7 kDa]). **H.** SDS-PAGE of purified MrpR WT, and the variants MrpR^G136E^, MrpR^Y304F^, MrpR^Y304A^, and MrpR^K169A^.

**Figure S3. Analysis of the asymmetric unit (ASU) of the MrpR crystal structure. A, B.** Overall fold of the two MrpR molecules found in the asymmetric unit (ASU) of the crystallographic analysis. One monomer is shown in rainbow color and one in grey. **C.** Surface representation of the two monomers present in the ASU shown in beige and light blue, respectively. **D.** Interacting residues in helices H’ and I of the two MrpR monomers are shown as orange and purple sticks. **E, F.** Zoom into the region of helices H’ and I highlighting the interacting residues found in the ASU of the MrpR crystal structure.

**Figure S4. Structural comparison of MrpR to known tyrosine recombinases. A.** N- and C-terminal domains of the MrpR crystal structure were individually overlayed with the structure of the Cre recombinase Cre (PDB-ID: 1Q3U) [1] as described in the main text and figure 5. **B-D.** Crystal structures of the tyrosine recombinases (**B**) Cre (PDB-ID: 1Q3U) [1]; (**C**) XerH (PDB-ID: 5JK0) [2], and (**D**) Lambda Int (PDB-ID: 1P7D) [3], all bound to DNA.

**Figure S5. Impact of G136E mutation on the MrpR protein. A.** Overall structure of MrpR in the putative DNA binding conformation. **B.** Overlay of MrpR crystal structure with the DNA-bound structure of the Cre recombinase (PDB-ID: 1Q3U) [1]. G136 is shown as a stick and highlighted. **C.** Zoom into the region of G136 located between α-helix G and H. Amino acid residues that might be affected by the G136E mutation are represented as sticks (L175, V177, F287). **D, E.** In silico Pymol mutation of G136 to a glutamate residue is illustrated in two putative structural rotamers of the glutamate side chain either facing the residue F287 (D) or L175/V177 (E).

**Figure S6. MrpR^G136E^ and MrpR^Y304F^ exhibit perturbations in higher order structure compared to MrpR.** **A.** The difference in HDX-MS between MrpR^G136E^ and MrpR or MrpR^Y304F^ and MrpR is displayed on the amino acid sequence of MrpR. Different tones of red indicate residues that incorporate more deuterium in the MrpR^G136E^ and MrpR^Y304F^ variants than in native MrpR indicating perturbations in their higher-order structure. The secondary structure of MrpR is schematically illustrated above. **B.** Representative peptides of MrpR exhibiting differences in HDX between MrpR (blue trace), MrpR^G136E^ (red trace) and MrpR^Y304F^ (green trace). Data represent the mean ± s.d. of n=3 replicates. **C.** The difference in HDX-MS between MrpR^G136E^ or MrpR^Y304F^ and MrpR (see panel A) was mapped onto the MrpR crystal structure. Hereby, each residue was color-coded as per the highest difference in HDX observed at any time-point. Red colors represent higher HDX in MrpR variants and grey colors no HDX difference compared to native MrpR. MrpR residues for which no peptides could be identified in HDX-MS are colored black. The G136E and Y304F sites of variation are shown as sticks colored in magenta.

**Figure S7. Hydrogen/deuterium exchange mass spectrometry of MrpR.** Each bar represents a peptide of MrpR that was identified in HDX-MS experiments. Peptides that could not be identified for MrpR^G136E^ or MrpR^Y304F^ variants due to the variations are highlighted in ocre and blue, respectively. The secondary structure of MrpR is depicted above (red boxes, α-helices; black arrows, β-strands). The residue-specific HDX of individual MrpR, MrpR^G136E^ and MrpR^Y304F^ residues in apo and DNA-bound states was calculated with DynamX 3.0 from peptides as follows: When any residue was covered by only a single peptide, the residue-specific deuterium uptake equals that of the whole peptide. In the case of overlapping peptides for any given residue, residue-specific deuterium uptake was determined by the shortest peptide covering that residue. Where multiple peptides were of the shortest length, the peptide with the residue closest to the peptide C-terminus was utilized. Numerical values for deuterium uptake by peptides and residue-specific HDX are provided in Supplemental Dataset.

**Figure S8. Structural comparison of catalytically important amino acid residues of tyrosine recombinases to those found in MrpR. A-D.** Comparison of V137/S138/G139 in MrpR (**A**) to R173 in Cre (**B**), R213 in XerH (**C**) and R212 in Lambda Int (**D**). **E-H.** Comparison of K169 in MrpR (**E**) to K201 in Cre (**F**), K239 in XerH (**G**) and K235 in Lambda Int (**H**). **I-L.** Comparison of N254 in MrpR (**I**) to H289 in Cre (**J**), H309 in XerH (**K**) and H308 in Lambda Int (**L**). **M-P.** Comparison of V257/R258 in MrpR (**M**) to R292 in Cre (**N**), R312 in XerH (**O**) and R311 in Lambda Int (**P**). **Q-T.** Comparison of V291 in MrpR (**Q**) to W315 in Cre (**R**), H335 in XerH (**S**) and H333 in Lambda Int (**T**). **U-X.** Comparison of Y304 in MrpR (**U**) to Y324 in Cre (**V**), Y344 in XerH (**W**) and Y342 in Lambda Int (**X**).

**Figure S9. *In vitro* recombinase assay. A.** Schematic overview of the *in vitro* recombination. The *attL* fragment (242 bp) is labeled with Cy5 (green) and the *attR* fragment (610 bp) is labeled with Cy3 (blue). The resulting hybrid *attP* and *attB* fragments indicative for DNA recombination have an expected size of 426 bp. **B.** Recombinase assay using the *attR* and *attL* regions of the SPβ phage assessing the recombinase activity of the purified MrpR WT protein and the MrpR variants MrpR^G136E^, MrpR^Y304F^, and MrpR^K169A^. Lanes marked with an asterisk (*) are reactions in which the labeled DNA fragments were exchanged by an unlabeled DNA fragment carrying no fluorescent dye.

**
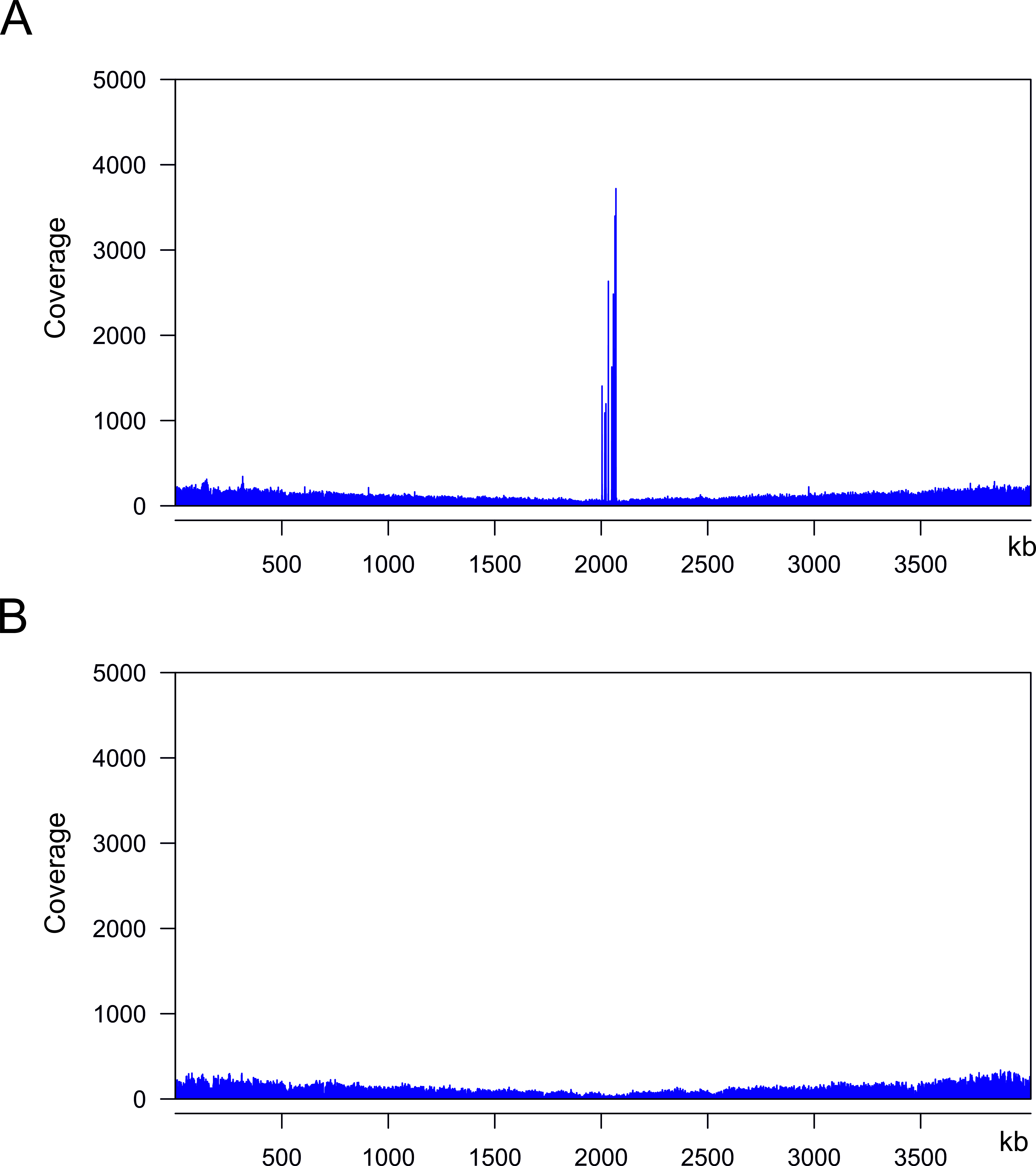
**

**Figure S10. Genome-wide profiling of MrpR DNA-binding in *B. subtilis*.** Mapping of ChAP-seq reads for MrpR binding to the whole genome of *B. subtilis* (**A**) MrpR-Strep of (**B**) untagged MrpR. The coverage of binding peaks (y axis) was plotted against the respective genomic regions of *B. subtilis* (x axis).

**Figure S11. Electrophoretic mobility shift assays (EMSAs) showing the DNA-binding activities of the MrpR wild-type protein and the MrpR^G136E^ variant.** **A, B.** Complex formation between the MrpR wild-type (WT) protein (**A**) and the MrpR^G136E^ variant (**B**) with the SPBRE-free *B. subtilis* DNA fragment *attR* (50 nM).

**Figure S12. Binding of DNA to MrpR and MrpR variants determined by isothermal titration calorimetry (ITC).** DNA of the *aimR* promoter region was added to the sample cell and titrated with purified MrpR WT (**A**, **C**) and MrpR variant proteins MrpR^G136E^ (**B**, **D**), MrpR^K169A^ (**E**, **G**) and MrpR^Y304F^ (**F**, **H**). Panels A, B, E and F show the thermograms for each ITC run, and panels C, D, G and H are the resulting fitted plots; the blue dots represent the binding enthalpies (ΔH) per injection versus ligand concentration. DP stands for differential power.

**Figure S13. DNA binding of MrpR, MrpR^G136E^ and MrpR^Y304F^ by HDX-MS.** **A.** The difference in HDX-MS between the DNA-bound and apo states of MrpR, MrpR^G136E^ and MrpR^Y304F^ is displayed on the amino acid sequence of MrpR. Different tones of blue indicate reduced deuterium incorporation in presence of DNA. The secondary structure of MrpR is schematically illustrated above. **B.** Representative peptides of MrpR and variants thereof exhibiting differences in HDX with respect to DNA. Data represent the mean ± s.d. of n=3 replicates. **C.** The difference in HDX-MS between the DNA-bound and apo state of native MrpR was projected onto a model of DNA-bound MrpR. The model was generated by superimposing the CAT domains of MrpR (residues 107-318) and DNA-bound Cre (PDB 1Q3U [1]; residues 130-341) followed by superposition of the MrpR CB domain (residues 1-106) onto that of DNA-bound Cre (residues 10-129). MrpR residues were color-coded as per the highest difference in HDX observed at any time-point. The G136E and Y304F sites of variation are shown as sticks colored in magenta.

**Table S1.** Primers used in the study.

| **Name** | **Features*** | **Purpose** |
| --- | --- | --- |
| PP073 | 5’-tacgTGATGCCTTCATCAACTAGA | Sequencing of *mrpR* deletion |
| PP312 | 5’-GGAACTAAGGTCGCAAATGG | Sequencing of *yokI* |
| PP318 | 5‘-AGCCTAGACGAGTTGGAAAG | Sequencing of *mrpR*/*mrpR*^G407A^ |
| PP319 | 5‘-GCAGCTGAGCGACTATAATC | Sequencing of *mrpR*/*mrpR*^G407A^ |
| PP326 | 5‘-GTCCCATAAGCCGGTTACAG | Sequencing of *yokI* |
| PP342 | 5‘-TTTCACCGGACAAAGCAACC | Sequencing of *yokI* |
| PP345 | 5‘-CCGCATTGCACATCTTTAGC | Sequencing of *yokI* |
| PP359 | 5’-TGTTTACCGGTGGATCGATG | Sequencing of *yosL* |
| PP360 | 5‘-CTATGTACGGCCTCCTTATC | Sequencing of *yosL* |
| PP366 | 5‘-ATA**GGATCC**ATAAGGAGATGTAAAATGGGG | Cloning of *yosL* with *Bam*HI |
| PP367 | 5‘-TAT**GAATTC**CTAATCGTTATAAGCCCCTG | Cloning of *yosL* with *Eco*RI |
| PP370 | 5‘-TAT**TCTAGA**AGGAGATATACATATGTTCAATAGT  GAGATTAAGGAA | Cloning of *mrpR-strep* |
| PP372 | 5‘-TATGGATCCATGTTCAATAGTGAGATTAAGGAA | Cloning of *mrpR-strep* |
| PP374 | 5‘-ATAAAGCTTAATGGTCGTCTCTTTTAGAC | Cloning of *mrpR-strep* |
| PP375 | 5‘-aaa**GAATTCt**tgtcaagtgaaggcgcgctatgcta  caatacagcttggtttaaaggaggaaacaatcATGTTCAATAGTGAGATTAAGGAA | Cloning of *mrpR*/*mrpR*^G407A^/*mrpR-Strep* with *Eco*RI |
| PP376 | 5‘-ttt**GGATCC**attacatgatcctccttaTTAAATGGT  CGTCTCTTTTAGAC | Cloning of *mrpR*/*mrpR*^G407A^ with *Bam*HI |
| PP388 | 5‘-ATA**GAATTC**GGCGAAACAGTGAGCCATAG | Cloning of *P_yosX_* with *Eco*RI |
| PP389 | 5‘-TAT**GGATCC**AATGAATCACCTCCTTAAAG | Cloning of *P_yosX_* with *Bam*HI |
| PP390 | 5‘-ATA**GAATTC**AGGATATTGCGGAAGAGGTG | Cloning of *P_aimR_* with *Eco*RI |
| PP391 | 5‘-TAT**GGATCC**ATTATTTCCCCTCATTTCTTTTTG | Cloning of *P_aimR_* with *Bam*HI |
| PPKK001 | 5‘-GACGAGCGGGATTTATCAGC | *sprA::aphA3* amplification from BKK21660 |
| PPKK002 | 5‘-CATATCACGAGGCAGTGTTG | *sprA::aphA3* amplification from BKK21660 |
| PPKK003 | 5‘-CATACACAACGAGCCCTGAC | *sprB::aphA3* amplification from BKK19820 |
| PPKK004 | 5‘-CAGTGAAGGAGGCCATTGAAG | *sprB::aphA3* amplification from BKK19820 |
| PPKK013 | TTT**GGATCC**ATTACATGATCCTCCTTATTACTTTTCGAACTGCGGGTGGCT | Cloning of *mrpR-strep* |
| TS037 | 5‘-tgaaatttcacctCTAatggtggtggtgatgatg  TTCGCCATTCAGGCTGCGCA | Sequencing of pAC5 and pAC7 based vectors |
| BR084 | 5‘-CCTGCTGTAATAATGGGTAGAAGGT | Sequencing of pAC5 and pAC7 based vectors |
| KG208 | 5‘-TTT**AGATCT**TCATTACTTGTACAGCTCGTCCATGC  CGA | Sequencing of pGP888 based vectors |
| JD024 | 5‘-GTTGGAGTTCGGAGTGAAAACAATTTTTTGATCT | Sequencing of pGP888 based vectors |
| IS096 | 5‘-GCAACTGTTGGGAAGGGCG | Sequencing of pAC5, pAC7 based vectors |
| MD082 | 5‘-CGTCAGCCGTAAACGCTTTT | Sequencing of *ganA* |
| MD083 | 5‘-ATGGCTGACACACCGGATTT | Sequencing of *ganA* |
| RH070 | 5’-AGA**TCTAGA**TCAATGGGGAAGAGAACCGC | *ermD* deletion cassette flankA |
| RH075 | 5’-TACGTTCCACTGCCAATACCTTAC | sgRNA targeting *ermD* |
| RH076 | 5’-aaacGTAAGGTATTGGCAGTGGAA | sgRNA targeting *ermD* |
| RH080 | 5’-tacgTACAATAGCGACGGAGAGTT | sgRNA targeting *cat* |
| RH081 | 5’-aaacAACTCTCCGTCGCTATTGTA | sgRNA targeting *cat* |
| RH082 | 5’-taggatccggccaacgaggccGGCAAGGAATAG  CAGTCCTAATGTATGT | Deletion of *cat* gene |
| RH083 | 5’-aaaaccttcttcaCATTTGATATGCCTCCTAAATTT  TTATCTAAAGTGA | Deletion of *cat* gene |
| RH084 | 5’-ggcatatcaaatgTGAAGAAGGTTTTTATATTAC  AGCTCCAGAT | Deletion of *cat* gene |
| RH085 | 5’-taggatccggccttattggccGAGATTTCCTGATA  TTGAACTTGCGGTT | Deletion of *cat* gene |
| RH112 | 5‘-CCATGTCGACCCGCTAGCTTTTTATTTTTAAAAAAT  TGTCAC | *ermD* deletion cassette flank A |
| RH113 | 5‘-AGA**TCTAGA**GTTCAGAGAATGATGCTTCC | *ermD* deletion cassette flank B |
| RH114 | 5’-AGCTAGCGGGTCGACATGGATGAGCGATG | *ermD* deletion cassette flank B |
| LC193 | 5‘-TTAA**GGTCTCC**CATGGGCGGGGCAGCTAGACGTA  TTGATC | Forward for pEJK9 |
| LC194 | 5´-TTAA**GGTCTCC**TCGAGATCGTTATAAGCCCCTGTA  ATTAATTTTGTAG | Reverse for pEJK9 |
| LC195 | 5´-TTAA**GGTCTCC**CATGGGCTTCAATAGTGAGATTA  AGGAAAAATATTTAG | Forward for pEJK11 and pEJK13 |
| LC196 | 5´-TTAA**GGTCTCC**TCGAGAATGGTCGTCTCTTTTAG  ACC | Reverse for pEJK11 and pEJK13 |
| LC226 | 5´-GCCGGAGATATGAACTACAAAGACATG | Forward for *P_aimR_* promotor |
| LC255 | 5´-[Cy3] GCTGCATTAGATGAAGAATTTAAAGAGC | Forward for *attR* site. Cy3 label at 5’ |
| LC256 | 5´-CTTTTGTAATAGCAGCCGATTGTAATG | Reverse for *attR* site |
| LC257 | 5´-CTATGATTCAGCGCACGGTGAACG | Forward for pEJK25 |
| LC258 | 5´- AGGGTTAAAACGTTACCG | Forward for pEJK25 |
| LC259 | 5´- AATGCAAGATGCGATTAATGAGGAAGAAG | Forward for pEJK29 |
| LC260 | 5´- GAATAAACTGCTCGATATTTG | Reverse for pEJK27 and pEJK29 |
| LC266 | 5´- AATGCAAGATTTTATTAATGAGGAAGAAG | Forward for pEJK27 |
| LC289 | 5´-[Dyomics781] CAGCATACTCCAATGCACTTCTTGC | Reverse for *aimR* site. Dyomics 781 label at 5’ |
| LC292 | 5´-GAATTTTAAAGAGATTACAAATACAAAAAATATG  TATAAAATAATTGACTCGTAATG | *aimR* promotor |
| LC293 | 5´-CATTACGAGTCAATTATTTTATACATATTTTTT  GTATTTGTAATCTCTTTAAAATTC | *aimR* promotor |
| EJK54 | 5´-GGATGAAGCAAGTAATGACTTAGATGAG | Forward for *attL* site |
| EJK55 | 5´-[Cy5] GACCATCCATTTGAAGCGATCC | Reverse for *attL* site. Cy5 label at 5’ |

***** Restriction sites are shown in bold letters, ribosome-binding sites and promoter elements are underlined; lower case letters indicate bases leading to a mutation/base exchange. Cy3, Cy3-labelled oligonucleotide; Cy5, Cy5-labelled oligonucleotide.

**Table S2.** Strains and plasmids

Strains

| **Name** | **Genotype** | **Usage/ construction^a^** | **Reference/**  **source** |
| --- | --- | --- | --- |
| *E. coli* | | | |
| DH10B | F^–^*mcr*A Δ(*mrr*-*hsd*RMS-*mcr*BC) φ80*lac*ZΔM15 Δ*lac*X74 *rec*A1 *end*A1 *ara*D139 Δ(*ara-leu*)7697 *gal*U *gal*K λ^–^*rps*L(Str^R^) *nup*G | Cloning | [4] |
| XL1-Blue | *recA1 endA1 gyrA96 thi-1 hsdR17 supE44 relA1 lac* [F*’* *proAB lacI*^q^ *Z*∆*M15* Tn*10* (*Tet^r^*)] | Cloning | Stratagene |
| BL21(DE3) | F^–^*omp*T *hsd*S_B_ (r_B_^–^, m_B_^–^) *gal dcm*(DE3) | Protein expression | NEB |
| *B. subtilis* | | | |
| 168 | *trpC2* | Laboratory strain | Laboratory collection |
| SP1 | Derivative of 168 | Laboratory strain | [5] |
| Δ6 | *trpC2* ΔSPβ (sublancin sensitive) Δ*skin* ΔPBSX Δprophage 1 *pks*::*cat* Δprophage 3 ΔICEBs1 | Derivative of 168 | [6] |
| CU1147 | *trpC2* SPβ c2 |  | [7] |
| TS01 | Δ6 *amyE*::(*P_mtlA_-comKS ermD*) | Derivative of Δ6 | [8] |
| TS03 | Δ6 Δ*cat* *amyE*::(*P_mtlA_-comKS ermD*) | Derivative of TS01 | This study |
| BKK19820 | *sprB*::*aphA3* | Derivative of 168 | [9] |
| BKE20080 | *yosL*::*ermC* | Derivative of 168 | [9] |
| BKE20790 | *mrpR*::*ermC* | Derivative of 168 | [9] |
| BKK21660 | *sprA*::*aphA3* | Derivative of 168 | [9] |
| KK001 | Δ6 *amyE*::(*P_mtlA_-comKS ermD*) SPβ c2 | SPβ c2 → TS01 | [10] |
| KK002 | Δ6 *amyE*::(*P_mtlA_-comKS*) SPβ c2 | Derivative of KK001 | [10] |
| KK004 | Δ6 *amyE*::(*P_mtlA_-comKS ermD*) *amyE::*(*P_alf4_-mrpR aphA3*) | Derivative of TS01 | [10] |
| KK137 | Δ6 *amyE*::(*P_mtlA_-comKS ermD*) SPβ | SPβ → TS01 | This study |
| KK009 | Δ6 *amyE*::(*P_mtlA_-comKS*) SPβ | Derivative of KK137 | This study |
| KK010 | Δ6 *amyE*::(*P_mtlA_-comKS ermD*) *amyE::*(*P_alf4_-mrpR*^G407A^ *aphA3*) | pRH166 → TS01 | This study |
| KK011 | Δ6 *amyE*::(*P_mtlA_-comKS*) SPβ c2 *amyE::*(*P_alf4_-mrpR aphA3*) | pRH167 → KK002 | This study |
| KK013 | Δ6 *amyE*::(*P_mtlA_ -comKS*) SPβc2  *amyE*::(*P_alf4_ -mrpR^G407A^ aphA3*) | pRH166 → KK002 | This study |
| KK014 | Δ6 *amyE*::(*P_mtlA_-comKS*) SPβ *amyE*::(*P_alf4_-mrpR*^G407A^ *aphA3*) | pRH166 → KK009 | This study |
| KK015 | Δ6 *amyE*::(*P_mtlA_-comKS*) SPβ *mrpR*::*ermC amyE::*(*P_alf4_-mrpR*^G407A^ *aphA3*) | BKE20790 → KK014 | This study |
| KK026 | Δ6 *amyE*::(*P_mtlA_-comKS ermD*) SPβ c2 *yosL*^+A1^ | Derivative of KK001 | This study |
| KK027 | Δ6 *amyE*::(*P_mtlA_-comKS ermD*) SPβ c2 *yosL*^T125A^ | Derivative of KK001 | This study |
| KK028 | Δ6 *amyE*::(*P_mtlA_-comKS*) SPβ c2 *yosL*::*ermC* | BKE20080 → KK002 | This study |
| KK031 | Δ6 *amyE*::(*P_mtlA_-comKS ermD*) *ganA*::(*xylR* *P_xylA_-yosL aphA3*) | pRH168 → TS01 | This study |
| KK032 | Δ6 *amyE*::(*P_mtlA_-comKS ermD*) *ganA*::(*xylR* *P_xylA_ aphA3*) | pGP888→ TS01 | This study |
| KK033 | Δ6 *amyE*::(*P_mtlA_-comKS*) SPβ c2 *ganA*::(*xylR* *P_xylA_-yosL aphA3*) | pRH168→ KK002 | This study |
| KK034 | Δ6 *amyE*::(*P_mtlA_-comKS*) SPβ c2 *ganA*::(*xylR* *P_xylA_ aphA3*) | pGP888 → KK002 | This study |
| KK056 | Δ6 *amyE*::(*P_mtlA_-comKS ermD*) SPβ c2 *yosL*^+A1^ *ganA*::(*xylR* *P_xylA_-yosL aphA3*) | pRH168 → KK026 | This study |
| KK058 | Δ6 *amyE*::(*P_mtlA_-comKS*) SPβ c2 *yosL*::*ermC ganA*::(*xylR* *P_xylA_-yosL aphA3*) | pRH168 → KK028 | This study |
| KK088 | Δ6 *amyE*::(*P_mtlA_-comKS*) SPβ c2 *amyE*::(*P_yosX_-lacZ aphA3*) | pRH170 → KK002 | This study |
| KK089 | Δ6 *amyE*::(*P_mtlA_-comKS*) SPβ c2 *amyE*::(*P_aimR_-lacZ aphA3*) | pRH171 → KK002 | This study |
| KK097 | Δ6 Δ*cat* *amyE*::(*P_yosX_-lacZ* *cat*) *ganA*::(*xylR* *P_alf4_-mrpR*^G407A^ *aphA3*) | pRH172 and pRH174 → TS03 | This study |
| KK098 | Δ6 Δ*cat* *amyE*::(*P_aimR_-lacZ cat*) *ganA*::(*xylR* *P_alf4_-mrpR*^G407A^ *aphA3*) | pRH173 and pRH174 → TS03 | This study |
| KK099 | Δ6 Δ*cat* *amyE*::(*P_yosX_-lacZ cat*) | pRH172 → TS03 | This study |
| KK100 | Δ6 Δ*cat* *amyE*::(*P_aimR_-lacZ cat*) | pRH173 → TS03 | This study |
| KK108 | Δ6 SPβ c2 *amyE*::(*P_alf4_-mrpR*^AAG505-507GCG^ *aphA3*) | pBP182 → KK002 | This study |
| KK118 | Δ6 SPβ c2 *amyE*::(*P_alf4_-mrpR*^TAT910-912TTT^) | pRH180 → KK002 | This study |
| KK119 | Δ6 SPβ c2 *amyE*::(*P_alf4_-mrpR*^TAT910-912GCG^ *aphA3*) | pRH181 → KK002 | This study |
| KK120 | Δ6 SPβ c2 *mrpR::ermC* *amyE*::(*P_alf4_-mrpR*^TAT910-912TTT^) | BKE20790 → KK118 | This study |
| KK121 | Δ6 SPβ c2 *mrpR*::*ermC amyE*::(*P_alf4_-mrpR*^TAT910-912GCG^ *aphA3*) | BKE20790 → KK119 | This study |
| KK124 | Δ6 *amyE*::(*P_mtlA_-comKS*) SPβ c2 *sprA*::*aphA3* | BKE21660 → KK002 | This study |
| KK125 | Δ6 *amyE*::(*P_mtlA_-comKS*) SPβ c2 *sprB::aphA3* | BKE19820 → KK002 | This study |
| KK138 | Δ6 SPβ c2 *mrpR*::*ermC amyE*::(*P_alf4_-mrpR*^AAG505-507GCG^ *aphA3*) | BKE20790 → KK108 | This study |
| KK139 | Δ6 Δ*cat* *amyE*::(*P_aimR_-lacZ cat*) *ganA*::(*xylR* *P_alf4_-mrpR aphA3*) | pKK005 → KK100 | This study |
| KK145 | Δ6 *amyE::*(*P_alf4_-mprR-Strep aphA3*) SPβ c2 | pKK012 → KK002 | This study |
| KK152 | Δ6 *amyE::*(*P_alf4_-mprR-Strep aphA3*) SPβ c2 *mrpR^G407A^*::*ermC* | BKE20790 →  KK145 | This study |
| KK167 | Δ6 *amyE::*(*P_alf4_-mprR^G407A^ aphA3*) SPβ c2 *mrpR^G407A^*::*ermC* | BKE20790 →  KK013 | This study |
| KK181 | Δ6 Δ*cat* *amyE*::(*P_yosX_-lacZ* *cat*) *ganA*::(*xylR* *P_alf4_-yopR aphA3*) | pKK005 → KK099 | This study |

^a^Arrows indicate construction by transformation or phage infection.

Plasmids

| **Name** | **Insert/purpose** | **Reference** |
| --- | --- | --- |
| pAC5 | No/translational promoter-*lacZ* fusions, complementation | [11] |
| pAC7 | No/translational promoter-*lacZ* fusions, complementation | [12] |
| pBP164 | *P_alf4_-lacZ cat/* transcriptional promoter-*lacZ* fusion | [13] |
| pET24d | For heterologous protein overproduction | Novagen |
| pGP888 | No/complementation | [14] |
| pJOE8999 | CRISPR-Cas9 engineering of *B. subtilis* | [15] |
| pKK005 | *aphA3 P_alf4_-mrpR P_xylA_ xylR*/expression of *mrpR* | This study |
| pKK012 | *P_alf4_-mprR-Strep*/expression of mprR-Strep and complementation | This study |
| pRH001 | Deletion of *ermD* gene from TS01 derivatives/ gRNA targeting *ermD* gene | This study |
| pRH004 | Deletion of the *cat* gene in TS001/gRNA targeting *cat* gene | This study |
| pRH005 | Deletion of the *cat* gene in TS001/gRNA targeting *cat* gene and recombination cassette | This study |
| pRH029 | Deletion of *ermD* gene from KK001 and KK137/gRNA targeting *ermD* gene and recombination cassette | This study |
| pRH166 | *P_alf4_-mrpR*^G407A^/expression of *mrpR*^G407A^ and complementation | This study |
| pRH167 | *P_alf4_-mrpR*/expression of *mrpR* and complementation | [10] |
| pRH168 | *xylR* *P_xylA_-yosL aphA3*/expression of *yosL* | This study |
| pRH170 | *P_yosX_-lacZ aphA3*/translational promoter-*lacZ* fusion | This study |
| pRH171 | *P_aimR_-lacZ kan*/transcriptional promoter-*lacZ* fusion | This study |
| pRH172 | *P_yosX_-lacZ cat*/transcriptional promoter-*lacZ* fusion | This study |
| pRH172 | *P_yosX_-lacZ cat*/transcriptional promoter-*lacZ* fusion | This study |
| pRH173 | *P_aimR_-lacZ cat*/transcriptional promoter-*lacZ* fusion | This study |
| pRH174 | *P_alf4_-mrpR*^G407A^ *xylR P_xylA_ aphA3*/expression of *mrpR*^G407A^ | This study |
| pRH180 | *P_alf4_-mrpR*^A911T^/expression of *mrpR*^A911T^ and complementation | This study |
| pRH181 | *P_alf4_-mrpR*^TAT910-912GCG^/expression of *mrpR*^TAT910-912GCG^ and complementation | This study |
| pRH182 | *P_alf4_-mrpR*^AAG505-507GCG^/expression of *mrpR*^AAG505-507GCG^ and complementation | This study |
| AL324 | pET24d derivative/Overproduction of C-terminal His_6_-tagged proteins | Laboratory collection Bange |
| pEJK9 | Heterologous overproduction of YosL-His_6_ | This study |
| pEJK11 | Heterologous overproduction of MrpR-His_6_ | This study |
| pEJK13 | Heterologous overproduction of MrpR^G136E^-His_6_ | This study |
| pEJK25 | Heterologous overproduction of MrpR^K169A^-His_6_ | This study |
| pEJK27 | Heterologous overproduction of MrpR^Y304F^-His_6_ | This study |
| pEJK29 | Heterologous overproduction of MrpR^Y304A^-His_6_ | This study |

**REFERENCES**

1. **Ennifar E, Meyer JEW, Buchholz F, Stewart AF, Suck D** (2003) Crystal structure of a wild‐type Cre recombinase– lox P synapse reveals a novel spacer conformation suggesting an alternative mechanism for DNA cleavage activation. Nucleic Acids Res 31: 5449-5460.
2. **Bebel A, Karaca E, Kumar B, Stark WM, Barabas O** (2016) Structural snapshots of Xer recombination reveal activation by synaptic complex remodeling and DNA bending. Elife 5.
3. **Aihara H, Kwon HJ, Nunes-Düby SE, Landy A, Ellenberger T (2003)** A Conformational Switch Controls the DNA Cleavage Activity of λ Integrase. Mol Cell 12: 187–198.
4. **Grant SG, Jessee J, Bloom FR, Hanahan D** (1990) Differential plasmid rescue from transgenic mouse DNAs into *Escherichia coli* methylation-restriction mutants. Proc Natl Acad Sci USA 874645-874649.
5. **Richts B, Hertel R, Potot S, Poehlein A, Daniel R, Schyns G, Prágai Z, Commichau FM** (2020) Complete genome sequence of the prototrophic *Bacillus subtilis* subsp. *subtilis* strain SP1. Microbiol Resour Announc 9: e00825-20.
6. **Westers H, Dorenbos R, van Dijl JM, Kabel J, Flanagan T, Devine KM, Jude F, Seror SJ, Beekman AC, Darmon E, Eschevins C, de Jong A, Bron S, Kuipers OP, Albertini AM, Antelmann H, Hecker M, Zamboni N, Sauer U, Bruand C, Ehrlich DS, Alonso JC, Salas M, Quax WJ** (2003) Genome engineering reveals large dispensible regions in *Bacillus subtilis*. Mol Biol Evol 20: 2076-2090.
7. **Rosenthal R, Toye PA, Korman RZ, Zahler SA** (1979) The prophage of SP beta c2dcitK1, a defective specialized transducing phage of *Bacillus subtilis*. Genetics 92: 721-739.
8. **Schilling T, Dietrich S, Hoppert M, Hertel R** (2018) A CRISPR-Cas9-based Toolkit for fast and precise *in vivo* genetic engineering of *Bacillus subtilis* phages. Viruses 10: 241.
9. **Koo BM, Kritikos G, Farelli JD, Todor H, Tong K, Kimsey H, Wapinski I, Galardini M, Cabal A, Peters JM, Hachmann AB, Rudner DZ, Allen KN, Typas A, Gross CA** (2017) Construction and analysis of two genome-scale deletion libraries for *Bacillus subtilis*. Cell Syst 4: 291-305.
10. **Kohm K, Floccari VA, Lutz VT, Nordmann B, Mittelstädt C, Poehlein A, Dragos A, Commichau FM, Hertel R** (2022) The *Bacillus* phage SPβ and its relatives: a temperate phage model system reveals new strains, species, prophage integration loci, conserved proteins and lysogeny management components. Environ Microbiol. 24: 2098-2118.
11. **Martin-Verstraete I, Débarboullié M, Klier A, Rapoport G** (1992) Mutagenesis of the *Bacillus subtilis* “-12, -24” promoter of the levanase operon and evidence for the existence of an upstream activating sequence. J Mol Biol 226: 85-99.
12. **Weinrauch Y, Msadek T, Kunst F, Dubnau D** (1991) Sequence and properties of *comQ*, a new competence regulatory gene of *Bacillus subtilis*. J Bacteriol 173: 5685-5693.
13. **Gundlach J, Herzberg C, Kaever V, Gunka K, Hoffmann T, Weiß M, Gibhardt J, Thürmer A, Hertel D, Daniel R, Bremer E, Commichau FM, Stülke J** (2017) Control of potassium homeostasis is an essential function of the second messenger cyclic di-AMP in *Bacillus subtilis*. Sci Signal 10: eaal3011.
14. **Diethmaier C, Pietack N, Gunka K, Wrede C, Lehnik-Habrink M, Herzberg C, Hübner S, Stülke J** (2011) A novel factor controlling bistability in *Bacillus subtilis*: the YmdB protein affects flagellin expression and biofilm formation. J Bacteriol 193: 5997-6007.
15. **Altenbuchner J** (2016) Editing of the *Bacillus subtilis* genome by the CRISPR-Cas9 system. Appl Environ Microbiol 82: 5421-5427.
